# Delaying or delivering: identification of novel *NAM-1* alleles that delay senescence to extend wheat grain fill duration

**DOI:** 10.1093/jxb/erab368

**Published:** 2021-08-18

**Authors:** Elizabeth A Chapman, Simon Orford, Jacob Lage, Simon Griffiths

**Affiliations:** 1 John Innes Centre, Norwich Research Park, Colney Lane, Norwich NR4 7UH, UK; 2 KWS-UK, 56 Church Street, Thriplow, Hertfordshire SG8 7RE, UK; 3 CSIRO Agriculture and Food, Australia

**Keywords:** Bulk segregant analysis, exome capture, forward genetics, grain fill, mutant, NAM, remobilization, senescence, staygreen, wheat

## Abstract

Senescence is a complex trait under genetic and environmental control, in which resources are remobilized from vegetative tissue into grain. Delayed senescence, or ‘staygreen’ traits, can confer stress tolerance, with extended photosynthetic activity hypothetically sustaining grain filling. The genetics of senescence regulation are largely unknown, with senescence variation often correlated with phenological traits. Here, we confirm staygreen phenotypes of two *Triticum aestivum* cv. Paragon ethyl methane sulfonate mutants previously identified during a forward genetic screen and selected for their agronomic performance, similar phenology, and differential senescence phenotypes. Grain filling experiments confirmed a positive relationship between onset of senescence and grain fill duration, reporting an associated ~14% increase in final dry grain weight for one mutant (*P*<0.05). Recombinant inbred line (RIL) populations segregating for the timing of senescence were developed for trait mapping purposes and phenotyped over multiple years under field conditions. Quantification and comparison of senescence metrics aided RIL selection, facilitating exome capture-enabled bulk segregant analysis (BSA). Using BSA we mapped our two staygreen traits to two independent, dominant, loci of 4.8 and 16.7 Mb in size encompassing 56 and 142 genes, respectively. Combining association analysis with variant effect prediction, we identified single nucleotide polymorphisms encoding self-validating mutations located in *NAM-1* homoeologues, which we propose as gene candidates.

## Teaser

Two staygreen mutants were identified for which grain fill duration and grain weight were enhanced. Independent amino acid substitutions within NAC subdomain IV of NAM-1 homoeologues are proposed as causative.

## Introduction

Monocarpic senescence is the terminal stage in wheat development, wherein 80% of leaf nitrogen is remobilized into developing grain (Buchanan-Wollaston, 2007). Genetic regulation of senescence involves significant transcriptional reprogramming enabling timely reallocation of resources. Plants with delayed senescence are described as ‘staygreen’, with cosmetic types resulting from impaired chlorophyll catabolism (Thomas and Ougham, 2014). Functional staygreen phenotypes are associated with enhanced or extended photosynthetic activity, conferring tolerance to heat, drought, and low nitrogen stress in multiple crops (Gregersen *et al.*, 2013; Thomas and Ougham, 2014).

Correlations between green canopy and grain fill duration of *r*=0.16–0.7 (*P*<0.01) (Pinto *et al.*, 2016; de Souza Luche *et al.*, 2017) support the potential breeding utility of staygreen traits, whereupon an extended grain filling period could increase grain size and enhance final grain yield. During grain fill Gelang *et al.* (2000) estimated the rate of grain weight increase as 0.96–1.25 mg d^−1^, with studies by Adu *et al.* (2011), Kitonyo *et al.* (2017), and Voss-Fels *et al.* (2019) suggesting staygreen traits have been selected over the past 50 years to sustain grain number improvement. For a *Triticum durum* cv. Trinkaria ethyl methane sulfonate (EMS) mutant, a 10 d delay in onset of senescence contributed to a 10–12% and 20% increase in thousand grain weight (TGW) and yield, respectively (Spano *et al.*, 2003). Under terminal heat stress, Kumari *et al.* (2007) confirmed the association between grain fill extension, TGW and yield improvement, with staygreen phenotype conferring a yield advantage. Modelling of wheat ideotypes using 2050 climate predictions weights staygreen traits highly, estimating associated yield benefits of 28–37% and 10–23% for Spanish and for Central and Eastern European growing regions, respectively (Senapati *et al.*, 2019), illustrating potential trait utility.

Phenology can confound senescence trait dissection (Bogard *et al.*, 2011; Camargo *et al.*, 2016). For example, of the stable senescence QTLs identified by Bogard *et al.* (2011), Naruoka *et al.* (2012), and Xie *et al.* (2016), several controlled anthesis date, encompassing genes *Ppd-D1*, *Vrn-B1*, and *Vrn-D3*. However, Verma *et al.* (2004), Naruoka *et al.* (2012) and Xie *et al.* (2016) reported phenology-independent senescence QTLs located on chromosomes 2DL, 2B, and 4A, with Naruoka *et al.* (2012) validating their QTL for ‘Green leaf area duration after heading’ using near isogenic lines. At the gene level, *NAM-B1* and *NAC-S* are known regulators of wheat senescence (Uauy *et al.*, 2006b; Zhao *et al.*, 2015). Both genes belong to the plant specific NAC transcription factor family, members of which are involved in hormone signalling, developmental pathways, and stress response (Olsen *et al.*, 2005; Podzimska-Sroka *et al.*, 2015).

Induced mutations are a vital source of novel variation, with 2250 mutant derived crop varieties released since 1940 (Ahloowalia *et al.*, 2004). In wheat, exome capture has been used to sequence the gene-encoding 2% of the genome to detect such novel variation, acting to reduce genetic complexity associated with its large genome size. (Henry *et al.*, 2014; Krasileva *et al.*, 2017). Genetic characterization of targeting induced local lesions in genomes (TILLING) populations and diverse material (Krasileva *et al.*, 2017; Alaux *et al.*, 2018) increases their applicability for use in forward and reverse genetic screens or functional characterization, accelerating gene discovery.

Here, we used exome capture-enabled bulk segregant analysis (BSA) to map novel staygreen alleles underpinning delayed senescence phenotypes of two independent *Triticum aestivum* cv. Paragon EMS mutants. We confirm results of the initial forward screen, and the relationship between onset of senescence and grain fill duration. Following repeated in-field phenotypic assessment of segregating recombinant inbred lines (RILs), we reduced senescence from a quantitative to qualitative trait enabling construction of staygreen and non-staygreen bulks. We used mapping-by-sequencing to identify two independent loci located on chromosomes 6A and 6D. Using variant effect prediction and single marker association analysis we refined these identified regions to likely gene candidates. Here, results converged upon self-validating, dominant mutations in homoeologous copies of known senescence regulator *NAM-B1* (Uauy *et al.*, 2006b), with marker development enabling selection of identified novel diversity.

## Materials and methods

### Plant material

#### Mutagenesis and initial screen

Seven thousand seeds of *Triticum aestivum* cv. Paragon (spring wheat) were treated with a 1% EMS solution for 16 h to obtain a 50% lethal dose for viability, in accordance with Rakszegi *et al.* (2010). Three thousand five hundred M_1_ seeds were sown to obtain M_1_ plants, with the surviving 3461 bagged to ensure self-fertilization. Two M_2_ seeds were sown per line, deriving ‘a’ and ‘b’ sibling lines (*n*=~6922) and advanced via multiple rounds of self-fertilization to the M_5_ generation. In spring 2006, ~6500 M_5_ lines were grown as single ear rows in 1 m^2^ plots at Church Farm, Norwich (52°38′N 1°10′E) (John Innes Centre, JIC). A single visual assessment of the 6500 M_5:6_ lines identified 18 early senescing and 43 staygreen mutants. Original data are available from: www.wgin.org.uk/wgin_2003-2008/index.php?area=Resources&page=results. In 2007, forward genetic screening of the *Triticum aestivum* cv. Paragon EMS population was repeated under low nitrogen conditions (100 kg N ha^−1^). Seed of the M_5:6_ generation (*n*=6500) was sown as single rows in 1 m^2^ plots. Phenotypic observations identified, or re-confirmed, differential senescence of ~80 lines (~1.2% in total). In 2008, 70 differentially senescing lines were included in a nitrogen use efficiency trial and grown as replicated 1 m^2^ plots receiving 20 kg N ha^−1^ and 240 kg N ha^−1^ (*n*=3). Fifty-four lines were subject to glasshouse experimentation, with lines 555a, 862a, 1389a, 2056a, and 2514a undergoing in-depth physiological characterization by Derkx *et al.* (2012). This study concerns genetic mapping of 1189a and 2316b, chosen for their environmentally stable, differential, staygreen phenotypes unconfounded by heading variation, and strong agronomic performance.

#### RIL population development

RIL populations segregating for senescence traits were developed for trait mapping purposes. 1189a and 2316b were crossed to parental *Triticum aestivum* cv. Paragon, and F_4_ populations developed through single seed descent (SSD): Paragon × 1189a (*n*=85), Paragon × 2316b (*n*=95). Additional RIL populations were developed segregating for senescence and heading date. 1189a and 2316b were crossed to a triple photoperiod insensitive (*Ppd-1a*) *Triticum aestivum* cv. Paragon near isogenic line (NIL), and F_3_ populations developed through SSD: Ppd × 1189a (*n*=96), Ppd × 2316b (*n*=96). Due to the use of two different triple *Ppd-1a* NILs, the source of the *Ppd-B1a* allele segregating amongst Ppd × staygreen RIL populations differs. The *Ppd-B1a* allele carried by the Ppd × 1189a RIL population originates from Sonora-64, whereas the Ppd × 2316b RIL population carries the Chinese Spring allele. NILs used share their *Ppd-A1a* and *Ppd-D1a* composition, and alleles originate from GS-100 and Sonora-64, respectively, as described in Bentley *et al.* (2011) and Shaw *et al.* (2012). Field multiplication of F_4_ (Paragon × staygreen) and F_3_ (Ppd × staygreen) seed was conducted in summer 2015, with RILs sown in 1 m^2^ plots (three rows, 40 cm spacing) in October 2014 (see Supplementary Fig. S1A).

### Phenotypic characterization

#### Field experimentation

In-field phenotyping of F_4_ Paragon × staygreen RIL populations was conducted between 2016 and 2018. Experiments were performed at Church Farm, Norwich (52°38′N 1°10′E), JIC. In 2016, 36 RILs per Paragon × staygreen population were sown as unreplicated 1 m^2^ spaced plots on 26 October 2015, alongside 36 Ppd × staygreen RILs. The selection of Ppd × staygreen RILs sown was informed by in-field phenotyping conducted in 2015, with nine RILs representing each ‘early’ or ‘late’ heading±staygreen combination selected per population. Ppd × staygreen RILs were also sown as 7 m^2^ yield plots in Meldreth, Cambridgeshire (52°05′N 0°00′W), KWS-UK, in mid-October 2015. Plots consisted of 10 rows drilled at a rate of 275 seeds m^−2^, and followed a latin-square design with cultivars Paragon, Soissons and KWS Santiago used as repeated checks.

In 2017 and 2018 experiments concerning Paragon × staygreen RILs consisted of replicated 6 m^2^ plots and incorporated additional RILs per population (2017, *n*=43, three replicates; 2018, *n*≥75, two replicates) and were sown on 26 October 2016 and 12 October 2017 (see Supplementary Fig. S1A). All experiments followed a randomized complete block design, with control plots (cv. Paragon, Soissons, 1189a, and 2316b mutant lines) randomly sown throughout. Seed used for field experiments was produced during multiplication of RILs in 2015 when the most recent source, but otherwise resulted from the preceding year.

The soil at Church Farm is described as sandy loam overlying alluvial clay, whereas the soil at the Cambridgeshire site is sandy clay loam. All experiments were rainfed but required supplemental irrigation in 2017. At Church Farm, seeds were dressed with Redigo Dieter (Bayer CropScience, Germany) and sown at a rate of 2750 seeds per 6 m^2^ (~300 plants m^−2^), with fertilizer applied over three occasions from late February to the end of April, totalling 214–228.5 kg N ha^−1^ and 62 kg SO_3_ ha^−1^. In Cambridgeshire nitrogen was applied when plants reached GS30 and GS32 and totalled 204 kg N ha^−1^. For both sites, all plots received standard fungicide and herbicide treatment. Mean daily temperature and rainfall data are provided in Supplementary Fig. S2 and were obtained from weather stations at Church Farm (52°38′N 1°10′E, Norwich) and Royston (52°2′N, 0°1′W, Cambridgeshire; http://dajda.net/).

#### Phenotypic assessment

Phenotyping was conducted at the plot level. Ear emergence (GS55) was scored when 50% of ears had emerged halfway from the flag leaf (Zadoks, *et al.*, 1974). Senescence was scored visually every 2–4 d after anthesis (daa) using a 0–100 scale (intervals of 5) based on Pask *et al.* (2012). Flag leaves were scored according to the proportion of leaf yellowing. A score of 5 represents leaf tip necrosis, whilst 100 indicates complete chlorosis or death (Pask *et al.*, 2012). Multiple flag leaves were assessed simultaneously to give a plot score. Peduncle senescence was scored in 2017 and 2018 and assessed as the percentage of yellow peduncles (top 3–5 cm) per plot based on three to four batches of 10 tillers.

To interpret senescence dynamics, senescence scores were plotted against thermal time in day °C from ear emergence (T_0_) to standardize for heading variation. For thermal time calculation, mean daily temperatures were calculated using minimum and maximum daily temperatures recorded by Church Farm weather station (location: 52°37′52.29″N, 1°10′23.57″E; Supplementary Fig. S2). Using time course senescence data corresponding to RILs and controls, senescence profiles were quantified and RILs classified into senescence types by deriving senescence metrics. Senescence metrics include mean senescence, onset, duration (from ear emergence or onset to terminal senescence) and thermal time to different leaf senescence scores. Onset and termination of senescence were considered the first time points for which senescence scores above 10 or 90, respectively, were first recorded. Calculation of time taken (in day °C) to reach specific senescence scores (TT30, TT40 …) is similar to MidS (50% senescence) (Christopher *et al.*, 2016), with senescence assumed to progress linearly between scoring points and time interpolated.

#### Grain filling experiments

To explore the relationship between senescence and grain filling traits, in 2017 and 2018 grain weight and moisture content were recorded for 1189a, 2316b, and cv. Paragon from anthesis to maturation. To standardize for developmental differences between tillers, ~50 ears per plot per genotype (2017, *n*=1; 2018, *n*=2) were tagged when 1–2 cm of peduncle became exposed. At 4- to 5-d intervals, starting from anthesis, five tagged ears per plot were sampled and sealed into a labelled ziplock bag to prevent moisture loss. Ears were then refrigerated whilst awaiting dissection (maximum 10 h post-sampling) and senescence of sampled plots scored. Ten grains per ear were dissected from the central region of the spike from floret positions 1 and 2 (the oldest grains; numbered according to Brinton and Uauy (2019)), placed into a single Eppendorf tube and weighed to determine fresh grain weight. With lids open, tubes were transferred to a 65 °C drying oven for grains to dry down, with oven door ajar to prevent condensation. After 48 h tubes were re-weighed to determine final dry grain weight and grain moisture content (%) calculated by dividing dry by fresh grain weight.

### Exome capture and sequencing

#### DNA extraction

Seeds of Paragon × 1189a and Paragon × 2316b F_4_ RIL populations, cv. Paragon, 1189a, and 2316b mutant parents were sown into individual cells of 96-well seed trays containing peat and sand and transferred to the glasshouse following 2–3 d of cold treatment. When plants reached the three-leaf stage, 5 cm of leaf tissue was harvested and collected into a 96-well collection tray (Qiagen, Germany, 19560) and material stored at −80 °C until required. DNA was extracted using a Qiacube (Qiagen) according to the QIAamp 96 DNA Qiacube HT Kit Protocol. DNA quality and quantity were analysed using a DS-11 spectrophotometer (Denovix, DE, USA), Qubit-4 fluorometer (dsDNA BR assay, Thermo Fisher Scientific Q32850) and by running a DNA sample on an agarose gel (1%) to detect high molecular weight DNA.

#### Exome capture

Following classification of F_4_ RILs into ‘staygreen’ and ‘non-staygreen’ types, DNA of selected individuals was pooled to form staygreen and non-staygreen bulks (see Fig. 3) and submitted for exome capture and sequencing. RILs for inclusion in bulks were selected based on within- and between-year concordance of senescence metrics, typically informed by a minimum of 2 years of phenotypic data. Details concerning bulk selection can be found in Supplementary Dataset S1. Provided quality control requirements specified by the sequencing provider were met, DNA of selected RILs was pooled, standardizing for DNA concentration to ensure equal RIL representation (1189a, *n*=17 for both bulks; 2316b, *n*=15 for staygreen, *n*=12 for non-staygreen) (see Supplementary Fig. S1B). Quality and quantity of bulked DNA was checked by running a DNA sample on an agarose gel (1%) and final DNA concentration determined using the Quibit-4 fluorometer (dsDNA BR assay).

Exome capture was used to sequence predicted gene-encoding regions (Krasileva *et al.*, 2017) of the four bulks and three parents (1189a, 2316b, and cv. Paragon). Library preparation, amplification, and sequencing were performed by Novogene (Hong Kong) using the SeqCapEZ probe set 140430_Wheat_TGAC_D14_REZ_HX1 for *Triticum aestivum* (Roche, Nimblegen, WI, USA), described by Krasileva *et al.* (2017). Libraries were sequenced using the HiSeq4000 platform (Illumina, CA, USA) producing 150 bp paired end reads. Sequencing read quality was analysed using FastQC (Andrews, 2010) with low quality sequences and adapter remnants removed using ‘sickle’ (version 1.2; paired end (pe) mode, default parameters (-q 20 -l 15) (Joshi and Fass, 2011).

#### Sequence alignment

Processed reads were aligned to *Triticum aestivum* cv. Chinese Spring RefSeq v1.0 (IWGSC *et al.*, 2018) using ‘BWA’ (version 0.7.17; command aln, default parameters (-n 4); sampe, default parameters (-n 10 -N 0)) (Li and Durbin, 2009). Aligned read pairs were retained and files converted from binary alignment/map (BAM) to sequence alignment/map (SAM) format and ordered, duplicates removed and read pairs indexed using ‘samtools’ (version 1.7; command view -f2, -S -h -u -b -o; sort -o; rmdup; index) (Li *et al.*, 2009). Variant calling was performed simultaneously for contrasting bulks and parents using ‘samtools’ (version 1.7; command mpileup, default parameters (-g -t DP, DV, DPR, INFO/DPR), producing a labelled output retaining read depth and quality information. Output files were parsed using bcftools (version 1.8) (Li and Barrett, 2011) using the multi-allelic calling mode (-mv) (Danecek *et al.*, 2014) generating multi-sample variant call format (.vcf) files. Variants assigned a quality score (‘QUAL’) above 20 were retained to improve accuracy, and positions with missing data removed to aid genotypic comparison using bcftools (version 1.8; command filter -I ‘%QUAL>20’ -e ‘FORMAT/GT[0–1]=‘./.’’) (Li and Barrett, 2011).

#### Bulk segregant analysis

To conduct BSA, multi-sample vcf files corresponding to each staygreen phenotypic classification were imported into RStudio (RStudio team, 2015), R version 3.5.2 (R Core Team, 2018), using the package ‘vcfR’ (Knaus and Grünwald, 2017). To identify variants enriched in staygreen versus non-staygreen bulks, single nucleotide polymorphism (SNP) indices were calculated for each position by dividing DV (the number of reads with the alternate allele) by DP (total read depth). Variants common to phenotypically contrasting bulks for which the SNP index=1 indicate varietal differences, occurring between cv. Paragon and Chinese Spring, and were removed. Remaining variants were filtered based on SNP index (staygreen>0.9, non-staygreen<0.1) and sequencing depth (DP>3). Mutations characteristic of EMS mutagenesis (G:A and C:T transitions) were prioritized for investigation and visualization in iGV (Integrated Genomics Viewer) (Robinson *et al.*, 2011). Variant enrichment across the genome was visualized by plotting ΔSNP index (SNP index_SG_ − SNP index_Non-SG_) using ggplot2 (Wickham *et al.*, 2018a).

#### Genotyping and genetic mapping

For SNPs of interest, homoeologue-specific kompetitive allele specific PCR (KASP) markers were designed manually and RIL populations genotyped. When designing primers, SNPs of interest plus 150 bases of upstream and downstream flanking sequence were extracted from the *Triticum aestivum* cv. Chinese Spring RefSeq v1.0 (IWGSC *et al.*, 2018) using a script supplied by Sophie Harrington. Extracted sequences were subject to BLAST searches against RefSeq v1.0 (IWGSC *et al.*, 2018) and Paragon assembly (Bian *et al.*, 2017) to detect homoeologous and varietal variants. Primer assay mixes contained 46 μl dH_2_O, 30 μl common primer (100 μM) and 12 μl of each tailed primer (100 μM). Genotyping assays were performed in 384-well format with a 2.5 μl KASP reaction volume consisting 14–18 ng DNA, 1.25 μl PACE 2× Mastermix (3crbio, UK), 1.25 μl dH_2_0 and 0.047 μl primer assay mix. KASP assays were performed using a hydrocycler or thermocycler, with cycling conditions as follows: 15 min at 94 °C, 10 cycles of 20 s at 94 °C, 60 s at 65–57 °C (decreasing by 0.8 °C per cycle), followed by 26–40 cycles of 20 s at 94 °C, 60 s at 57 °C. PCR cycling conditions required optimization for some markers. KASP markers developed are listed in Supplementary Table S1 (1189a) and Supplementary Table S2 (2316b). Fluorescence was measured using a Pherastar plate reader (BMG Labtech, Germany) and data analysed using KlusterCaller software (version 4.1; LGC Genomics, UK).

Genetic maps for RIL populations were constructed from KASP genotyping data using the ‘Kosambi’ mapping function, specifying a LOD score of 6 in MapDisto v2.0 (Heffelfinger *et al.*, 2017). Loci were initially ordered using ‘Automap’ and ‘Find group’ functions and loci with missing data removed. Loci were reordered using ‘ripple order’, ‘check inversions’, and ‘order sequence’ functions with optimal order chosen based on shortest computed length and greatest increase in LOD score. Genetic maps were drawn using MapChart (Voorrips, 2002).

Single marker analysis was performed to identify peak marker-trait associations using the package ‘AssocTests’ (Wang *et al.*, 2017). To correct for multiple testing, outputted *P*-values were adjusted using the Holm correction. To determine if identified SNPs were causal variant effect prediction (McLaren *et al.*, 2016) was performed and SIFT score outputted (0=deleterious, 1=tolerable). To confirm the Mendelian mode of inheritance of staygreen traits, phenotype×genotype plots were constructed for peak markers using the package ‘r/qtl’ (Arends *et al.*, 2010) and replotted using ggplot2 (Wickham *et al.*, 2018a).

### Data analysis

Data analysis was performed using R (version 3.5.2) (R Core Team, 2018) in RStudio (RStudio team, 2015) and data manipulated using the packages ‘data.table’ (Dowle *et al.*, 2019), ‘dplyr’ (Wickham *et al.*, 2018*b*), ‘plyr’ (Wickham, 2015) and ‘tidyr’ (Wickham *et al.*, 2019). Senescence metrics were derived from raw senescence phenotyping data in the absence of spatial correction and means calculated per line when replicated. Analysis of senescence and grain filling profiles of individual lines or groups was conducted using linear mixed modelling using the packages ‘lme4’ (Bates *et al.*, 2019) and ‘lmerTest’ (Kuznetsova *et al.*, 2017). Data for each year/site were analysed separately. When conducting time course senescence or grain filling analysis two models were used, one to determine the overall difference, the other for specific time points:


$$\begin{array}{l} TimeCourse\;<-\;lmer(Phenotype\;∼\;Date\;\\ +\;Genotype\;+\;\left(1|Plot:Rep\right)) \end{array}$$

$$\begin{array}{l} TimePoint\;<-\;lmer(Phenotype\;∼\;Date/Genotype\;\\ +\;\left(1|Plot:Rep\right)) \end{array}$$

When analysing differences in senescence metrics once genotyped, a complete linear mixed model was initially applied, detailed below:


$$\begin{array}{l} SenescenceModelling\;<-\;lmer(SenMetric\;∼\;Rep\;+\;Row\;\\ +\;Column\;+\;Variety/Population/\\ \left(\left(NAM.A1\;+\;NAM.D1\right)*GS55\right)\;+(1|Population:Genotype)) \end{array}$$

In all cases, fixed-effect terms for retention were identified through ANOVA, with non-significant terms (*P*>0.05) dropped in an iterative manner. Significance of random terms was assessed using command ‘rand(model)’ with term retained when significant (*P*<0.05). Goodness of fit was visually assessed using QQ-plots and whether residuals versus fitted values demonstrated a normal distribution. Tukey *post hoc* tests were performed using the package ‘lsmeans’ (Lenth, 2018) to determine the significance of differences between genotypic groups, or individual lines, both overall and at specific time points. Graphs were constructed using ‘ggplot2’ (Wickham *et al.*, 2018a).

## Results

### Identification of staygreen mutants

**Fig. 1.**
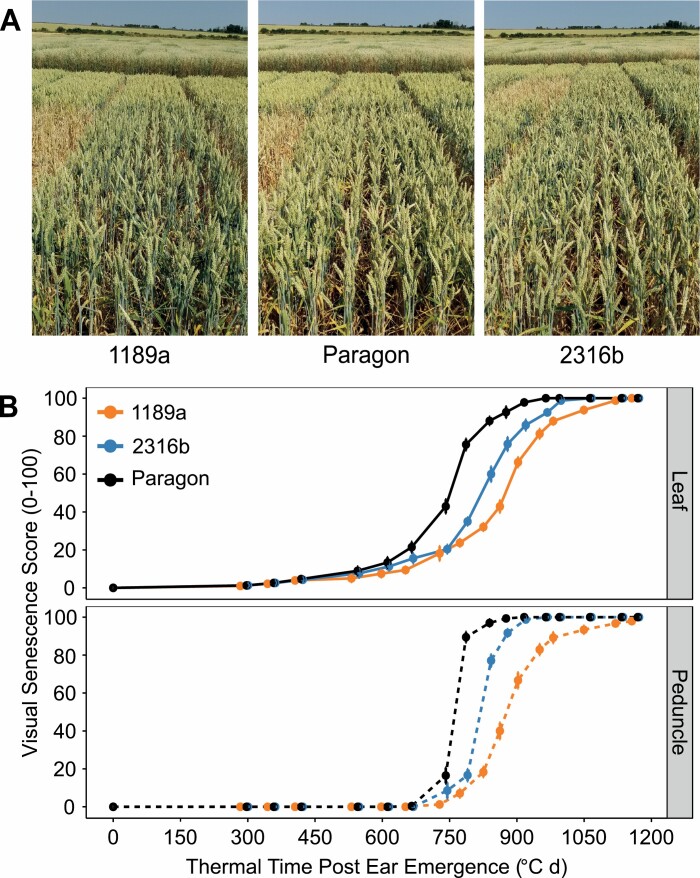
Identification and confirmation of two staygreen mutants. (A) Visual differences in senescence of 1189a (left) and 2316b (right) compared with cv. Paragon (centre) relate to variation in onset. Plots photographed 5 July 2018, GS55 within ±1 day. (B) Progression of flag leaf (top) and peduncle (bottom) senescence of 1189a, 2316b, and cv. Paragon in 2017. Senescence was scored visually using a 0–100 scale two to four times per week from ear emergence (GS55), with scoring dates converted to thermal time (day °C). Mean ±SEM, *n*≥12. Lines shown: cv. Paragon (black), 1189a (orange), 2316b (blue).

Six thousand five hundred M_5:6_ generation *Triticum aestivum* cv. Paragon EMS mutant lines were phenotyped under field conditions at JIC between 2006 and 2007. Approximately 80 lines (~1.2%) displayed differential senescence phenotypes, with staygreen phenotypes of 1189a and 2316b unconfounded by heading-date variation (Fig. 1A). To confirm these results detailed phenotyping of 1189a and 2316b was conducted between 2016 and 2018, identifying staygreen phenotypes as characterized by delays in the onset of senescence. Time course leaf and peduncle senescence profiles of 1189a and 2316b differed significantly when compared with cv. Paragon (*P*<0.05) (Fig. 1B; Supplementary Fig. S3). Compared to cv. Paragon leaf senescence of 1189a and 2316b is initiated 6–10 and 3–4 d later, respectively, whilst senescence rate is unaffected (Table 1), with a similar pattern observed for peduncle senescence (Fig. 1B; Supplementary Fig. S3).

**Table 1.** Senescence pairwise comparison (*P*-value, staygreen mutant versus cv. Paragon)

	1189a				2316b			
**Year**	Ear emergence (GS55) (dd/mm ±SD)	Onset (dd/mm)	Leaf	Peduncle	Ear emergence (GS55) (dd/mm ±SD)	Onset (dd/mm)	Leaf	Peduncle
2016	08/06±1.1 (+1)	29/07 (+ 10)	0.11	—	08/06±1.9 (+1)	23/07 (+ 4)	0.0005	—
2017	30/05±1.4 (+1)	06/07 (+ 6)	< 0.0001	< 0.0001	29/05±0.8 (=)	04/07 (+ 4)	< 0.0001	0.002
2018	01/06±0.9 (+1)	07/07 (+ 6)	< 0.0001	< 0.0001	31/05±1.2 (=)	03/07 (+ 3)	0.08	0.048

Results of Tukey *post hoc* tests comparing overall leaf and peduncle time course senescence. Date of ear emergence (GS55) and onset of leaf senescence with days difference relative to cv. Paragon in parentheses. Peduncle senescence was unscored in 2016.

### Extended grain fill duration of staygreens

Maintenance of green leaves has been associated with extending grain fill duration and grain weight enhancement (Wiegand and Cuellar, 1981; Gelang *et al.*, 2000; Bogard *et al.*, 2011). To test this, we recorded grain weight and moisture content to determine grain fill duration of 1189a, 2316b, and cv. Paragon in 2017 and 2018.

**Fig. 2.**
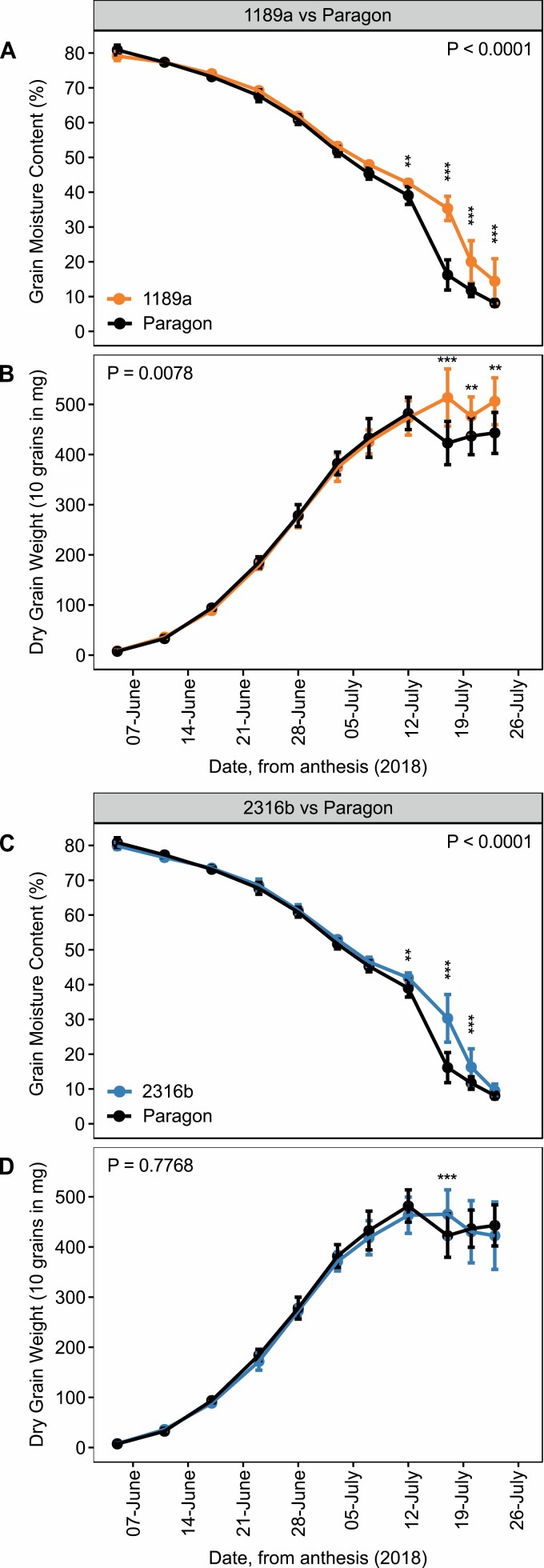
Grain filling dynamics of 1189a and 2316b are altered compared with cv. Paragon. Grain fill duration, determined by the reduction in grain moisture content, is longer for 1189a (A) and 2316b (C) (*P*<0.0001). Grain filling extension is associated with greater dry grain weight accumulation and final grain weight recorded for 1189a (B) (*P*<0.001), but not 2316b (D) (*P*>0.2). In 2018, grain moisture content (%) and dry weight (10 grains in mg) were recorded at 3–6-d intervals starting from anthesis (5 June 2018). Mean ±SD, *n*=4–5, two plots per line. *P*-values shown on the figure represent overall differences (ANOVA). Pairwise differences indicated at corresponding time points, *P<0.05, **P<0.01, ***P<0.001 (Tukey *post hoc* test). Corresponding senescence profiles plotted in Supplementary Fig. S5. Grain filling experiment was repeated in 2018 following results obtained in 2017 (Supplementary Fig. S5), with Supplementary Table S3 reporting statistics for both.

Grain moisture content declined more slowly for 1189a and 2316b when compared with cv. Paragon (*P*<0.0001) (Fig. 2A, C; Supplementary Fig. S4). Forty-two days after anthesis (daa) grain moisture content of our staygreens was 17–20% greater compared to cv. Paragon, *P*≤0.007 (Fig. 2A, C; Supplementary Fig. S4; Supplementary Table S3). Subsequently, grain moisture content of 1189a remained elevated (*P*<0.001), suggesting an extended time to reach grain maturity (Fig. 2A; Supplementary Fig. S4). Between 48 and 52 daa, differences in grain moisture content between 2316b and cv. Paragon were not significant, with time to grain maturation similar (*P*>0.2) (Fig. 2C; Supplementary Fig. S4). Relative to cv. Paragon, differences in grain moisture for 1189a and 2316b were significant at four and three time points, respectively (Supplementary Table S3), reflecting the differences in onset of senescence (Table 1; Supplementary Figs S4, S5). Extended duration of grain filling observed for our staygreens did not consistently increase final dry grain weight. Dry grain weight accumulation for 2316b matched cv. Paragon in both years (*P*>0.1) (Fig. 2D; Supplementary Fig. S4), but was greater for 1189a in 2018 (*P*<0.001) (Fig. 2B). In 2017, between 37 and 47 daa dry grain weights for 1189a and 2316b were greater relative to cv. Paragon (*P*<0.05) (Supplementary Fig. S4), but these differences did not contribute to a significant increase in final grain weight as recorded on 23 July (*P*>0.05) (Supplementary Table S3). Conversely, for 1189a the significant differences in dry grain weight recorded between 42 and 48 daa (*P*<0.01) did translate to an 11–14.4% increase in final dry grain weight (*P*<0.001) in 2018 (Fig. 2B; Supplementary Table S3).

### Identifying senescence extremes

To genetically map our staygreen traits we adopted a complexity reduction BSA approach. Repeated in-field phenotyping of Paragon × 1189a and Paragon × 2316b RILs enabled their accurate classification into senescence types. For each experiment senescence profiles of individual RILs were quantified by deriving senescence metrics. RILs were considered ‘non-staygreen’ or ‘staygreen’ based on whether their mean phenotypic scores were lower or higher when compared with cv. Paragon, or respective staygreen parent. RILs for which senescence metrics fell between parental values were classified manually based on concordance of metrics able to be readily classified, alongside parental senescence curve comparison. To assess phenotypic stability of RILs inter-year comparisons were performed, with bulk selections guided by a minimum of one, but typically two, years of data for 1189a and 2316b, respectively. Therefore, despite some of the additional RILs grown in 2018 displaying senescence phenotypes towards the extreme ends, these went largely unselected due to multi-year data providing greater confidence in our selections. Details of RIL classification and bulk selections are supplied in Supplementary Dataset S1.

**Fig. 3.**
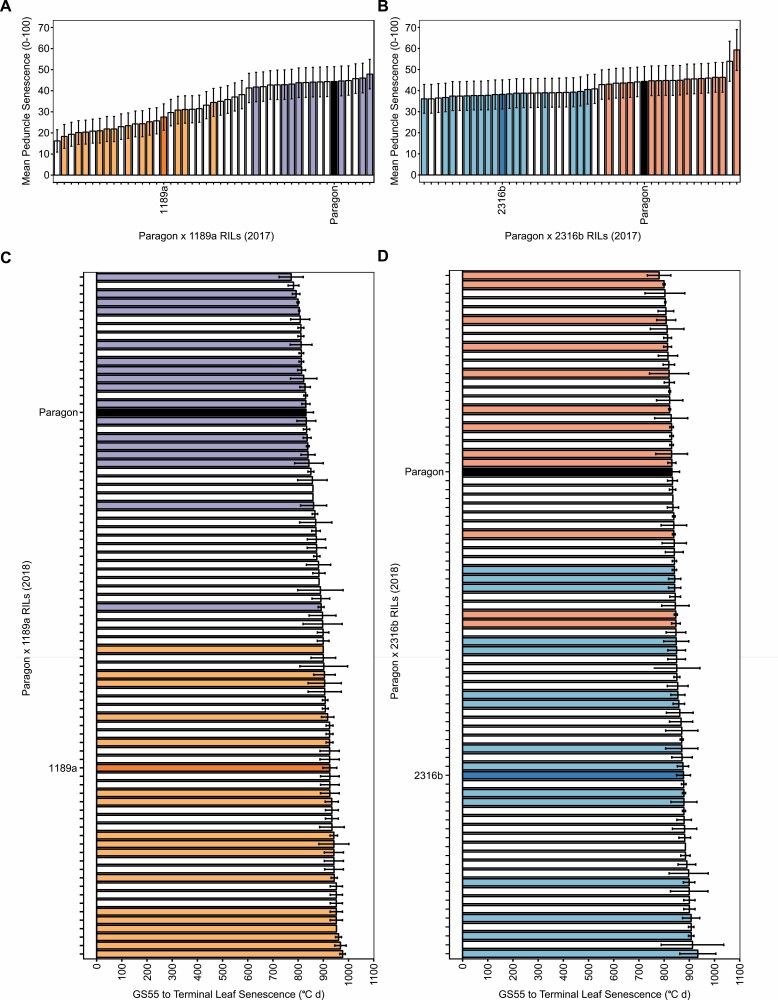
Senescence variation amongst F_4_ RIL populations, highlighting RILs included in bulks. (A, B) Mean peduncle senescence scores for Paragon × 1189a (A) and Paragon × 2316b (B) F_4_ RILs grown in 2017; mean ±SD, *n*=3. (C, D) Duration of leaf senescence (from ear emergence) for Paragon × 1189a (C) and Paragon × 2316b (D) F_4_ RILs grown in 2018; mean ±SD, *n*=2. Coloured bars represent parents and RILs included in bulks: 1189a (orange), ‘staygreen’ (light orange, *n*=17), ‘non-staygreen’ (light purple, *n*=17); 2316b (dark blue), ‘staygreen’ (light blue, *n*=15), ‘non-staygreen’ (red, *n*=12); Paragon (black). Classification and selection of RILs guided by multiple senescence metrics with intra- and inter-year comparisons performed.

Senescence progression was highly dynamic between years, and when classifying and selecting RILs the discriminatory power of senescence metrics varied, with reference to multiple metrics preferred. In 2017, mean peduncle senescence scores were most discriminative, opposed to duration of leaf senescence in 2018 (Fig. 3). The metric TT70 consistently discriminated senescence variation, with large differences always visible between parental lines and segregating RILs (Supplementary Figs S6, S7).

### 1189a and 2316b staygreen traits are located on chromosomes 6A and 6D

Exome capture sequencing reads were aligned to the *Triticum aestivum* cv. Chinese Spring RefSeq v1.0 (IWGSC *et al.*, 2018) and variants identified (Supplementary Fig. S1). Coverage of high confidence exons according to IWGSC gene annotation v1.1 (Alaux *et al.*, 2018; IWGSC *et al.*, 2018) ranged from ~35% to 43%, equating to over 131 million positions for each parental line or bulk. For each exome capture sample, a read depth ≥20 was recorded for 80–90 million positions, not exclusive to exons. Of these positions ~57 000–68 000 were SNPs, with one-third being characteristic EMS G:A or C:T transitions, with SNPs identified within the cv. Paragon sample being varietal (Supplementary Table S4).

Mapping traits using BSA aims to identify variants that are both enriched and depleted between contrasting bulks across genetic region(s) (Michelmore *et al.*, 1991). To standardize for variation in read depth, a SNP index is calculated by dividing DV by DP (number of reads for the alternative allele/total read depth). SNPs associated with delayed senescence phenotypes should be present in most reads within ‘staygreen’ bulks (SNP index=1) but absent in the ‘non-staygreen’ bulk (SNP index=0; ΔSNP index=1). Variants for which SNP index=1 across both bulks are varietal (ΔSNP index=0).

**Fig. 4.**
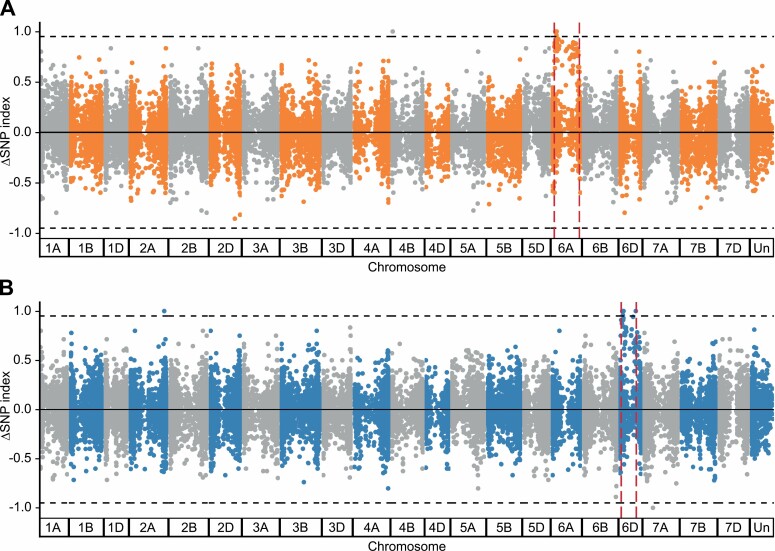
Bulk segregant analysis maps 1189a and 2316b staygreen traits to two independent loci. Calculation of ΔSNP index (SNP index_SG_ − SNP index_Non-SG_) identifies SNPs characteristic of EMS-mutagenesis that are enriched across single regions of chromosome 6A for 1189a (A) and chromosome 6B for 2316b (B). SNPs plotted are G:A and C:T transitions (1189a, *n*=37 495; 2316b, *n*=38 947) against physical position of *Triticum aestivum* cv. Chinese Spring RefSeq v1.1 (IWGSC *et al.*, 2018).

Upon removal of varietal SNPs, 37 495 and 38 947 transition type SNPs (DP≥5) were retained across the 1189a and 2316b bulks, respectively. Filtering SNPs based on SNP index (non-staygreen ≤0.05 and staygreen ≥0.95) identified independent regions on chromosomes 6A and 6D enriched for 1189a and 2316b alleles, respectively (Fig. 4). For 1189a and 2316b, six SNPs on chromosome 6A and 10 SNPs on chromosome 6D were found to be completely enriched (ΔSNP index=1), respectively (Tables 2, 3). Additional positions for which a ΔSNP index=1 include variants located on chromosome 4B for 1189a, and 2A, 2D, and 7D for 2316b, but none were predicted to be deleterious, and in the absence of enriched flanking SNPs (ΔSNP index>0.9) these regions were not considered to be of interest (Tables 2, 3).

**Table 2.** Bulk segregant analysis maps the 1189a allele to a chromosomal region on 6A encoding a mutation in NAM-A1

Position^a^	SNP	∆SNP index	Effect^b^	∆AA^b^	SIFT^b^	Gene^c^	Function^c^
chr4B_17232065	C:T	1.000	Intronic	—	—	TraesCS4B02G024000	Argonaute (*T. urartu*)
chr6A_46782541	G:A	0.851	Missense	R/C	0	TraesCS6A02G076600	Uncharacterized
chr6A_55741222	G:A	0.888	Intronic	—	—	TraesCS6A02G087400	HBP-1a transcription factor (*T. urartu*)
chr6A_57668862	G:A	0.876	Missense	A/T	0	TraesCS6A02G099900	Snare region anchored in the vesicle membrane C-terminus
**chr6A_67373891**	**G:A**	**1.000**	**Missense**	**A/T**	**0**	**TraesCS6A02G099900**	**ZIP zinc transporter**
**chr6A_76523438**	**G:A**	**1.000**	**intronic**	**—**	**—**	**TraesCS6A02G107700**	**UDP-***N***-acetylglucosamine–peptide ***N***-acetylglucosaminyltransferase SPINDLY (***T. urartu***)**
**chr6A_77099433**	**G:A**	**1.000**	**Missense**	**T/I**	**0**	**TraesCS6A02G108300**	**NAM-A1 (no apical meristem)**
**chr6A_80699949**	**G:A**	**1.000**	**Upstream**	**—**	**—**	**TraesCS6A02G111400**	**Cycloartenol synthase (***T. urartu***)**
**chr6A_84072498**	**G:A**	**1.000**	**Intronic**	**—**	**—**	**TraesCS6A02G114100**	**bHLH95 (***T. urartu***)**
chr6A_93819244	G:A	0.964	Missense	H/Y	1	TraesCS6A02G121600	DUF1644
chr6A_94068104	G:A	0.933	Missense	G/S	0	TraesCS6A02G121900	Cellulose synthase
chr6A_96601065	G:A	0.943	Synonymous	A	—	TraesCS6A02G123000	CASP-like protein 16 (*Zea Mays*)
chr6A_98048315	G:A	1.000	Missense	P/L	0	TraesCS6A02G124200	Cytosine methyltransferase (*Hordeum vulgare*)
chr6A_104309957	G:A	0.892	Intergenic	—	—	—	—
chr6A_116709274	G:A	0.915	Missense	A/T	0.02	TraesCS6A02G141800	E3-ubiquitin ligase (*Aegilops tauschii*)
chr6A_137292502	G:A	0.923	Intergenic	—	—	—	—
chr6A_214559812	C:T	0.898	Missense	S/N	0	TraesCS6A02G184000	Leucine-rich repeat receptor-like protein kinase (*Zea mays*)
chr6A_297671234	G:A	0.806	Missense	V/I	0.72	TraesCS6A02G197400	Uncharacterized
chr6A_371735058	G:A	0.852	Synonymous	A	—	TraesCS6A02G208100	Pentatricopeptide repeat family protein (*Zea mays*)
chr6A_456779221	G:A	0.885	Missense	A/V	0.51	TraesCS6A02G245500	U-box domain-containing protein (*Aegilops tauschii*)
chr6A_458220665	G:A	0.893	Intronic	—	—	TraesCS6A02G247100	BEACH domain containing protein (*Aegilops tauschii*)
chr6A_459076102	G:A	0.875	Missense	P/S	0.35	TraesCS6A02G247200	GRAS transcription factor (*Zea mays*)
chr6A_479267518	G:A	0.823	Missense	T/I	0	TraesCS6A02G257000	Uncharacterized
chr6A_504123510	G:A	0.883	Intergenic	—	—	—	—
chr6A_517559399	G:A	0.879	Synonymous	R	—	TraesCS6A02G285800	Ethylene receptor (*Aegilops tauschii*)

Bold text indicates the genetic region reporting ΔSNP index=1.

^*a*^ IWGSC RefSeq v1.0 co-ordinates (IWGSC *et al.*, 2018).

^*b*^ Variant effect prediction (McLaren *et al.*, 2016).

^*c*^ IWGSC annotation v1.1 (Alaux *et al.*, 2018).

**Table 3.** Bulk segregant analysis maps the 2316b allele to a chromosomal region on 6D encoding a mutation in NAM-D1.

Position^a^	SNP	∆SNP index	Effect^b^	∆AA^b^	SIFT^b^	Gene^c^	Function^c^
chr2A_732959676	C:T	1.000	Intergenic	—	—	—	—
chr2D_15472677	C:T	1.000	Missense	A/T	0.38	TraesCS2D02G042900	NBS-LRR resistance like protein (*Hordeum vulgare*)
chr3A_8326626	C:T	0.833	Intronic	—	—	TraesCS3A02G009200	NB-ARC domain
chr3B_12565097	C:T	0.875	Intergenic	—	—	—	—
chr3D_588999854	G:A	0.833	Splice region, synonymous	—	—	TraesCS3D02G498000	Protein tyrosine kinase
chr6D_36628312	G:A	0.909	3′ UTR	—	—	TraesCS6D02G072100	PR17c precursor (*Hordeum vulgare*)
chr6D_42295410	C:T	1.000	Intergenic	—	—	—	—
chr6D_50958137	G:A	0.875	Downstream	—	—	TraesCS6D02G085600	Uncharacterized
**chr6D_57044844**	**C:T**	**1.000**	**Intergenic**	**—**	**—**	**—**	**—**
**chr6D_60487321**	**C:T**	**1.000**	**Missense**	**G/E**	**0**	**TraesCS6D02G096300**	**NAM-D1 (no apical meristem)**
**chr6D_65341694**	**C:T**	**1.000**	**Missense**	**G/S**	**0.56**	**TraesCS6D02G101900**	**F-box domain**
chr6D_72022762	C:T	0.960	Intronic	—	—	TraesCS6D02G107900	SNF2 family N-terminal domain
chr6D_79076045	G:A	0.909	Missense	A/T	0.32	TraesCS6D02G112400	Diaminopimelate decarboxylase, chloroplastic (*T. urartu*)
chr6D_84222670	G:A	0.929	Downstream	—	—	TraesCS6D02G119000	Tyrosine-sulfated glycopeptide receptor 1-like (*Aegilops tauschii*)
chr6D_86733052	G:A	1.000	Intronic	—	—	TraesCS6D02G122300	GDP-fucose protein *O*-fucosyltransferase
chr6D_110872402	G:A	0.756	Synonymous	—	—	TraesCS6D02G141300	FGGY carbohydrate kinase domain-containing protein (*Aegilops tauschii*)
chr6D_119464714	G:A	0.755	Intergenic	—	—	—	—
chr6D_139310981	G:A	0.817	Intronic	—	—	TraesCS6D02G161000	Lycopene β-cyclase (*T. urartu*)
chr6D_140102996	G:A	0.833	Intronic	—	—	TraesCS6D02G161800	Aminotransferase (*Zea mays*)
chr6D_148079666	G:A	0.790	Missense	P/S	0.01	TraesCS6D02G166500	Glycosyltransferase (*Aegilops tauschii*)
chr6D_264681596	G:A	0.815	Missense, splice region	G/D	0	TraesCS6D02G192000	Hypothetical methyltransferase
chr6D_292379919	G:A	0.941	Intronic	—	—	TraesCS6D02G206900	S1 RNA binding domain
chr6D_352357451	C:T	1.000	5′ UTR	—	—	TraesCS6D02G248900	—
chr6D_363004043	G:A	1.000	Synonymous	—	—	TraesCS6D02G256800	GPI-anchored protein (*Aegilops tauschii*)
chr7D_8178083	C:T	0.833	Synonymous	—	—	TraesCS7D02G018200	NBS-LRR type resistance
chr7D_583986610	G:A	1.000	Intronic	—	—	TraesCS7D02G470700	—

Bold text indicates the genetic region reporting ΔSNP index=1.

^*a*^ IWGSC RefSeq v1.0 co-ordinates (IWGSC *et al.*, 2018)

^*b*^ Variant effect prediction (McLaren *et al.*, 2016)

^*c*^ IWGSC annotation v1.1 (Alaux *et al.*, 2018)

### 1189a and 2316b staygreen traits are underpinned by mutations in *NAM-1*

To determine if SNPs identified as enriched by BSA are causative, variant effect prediction was conducted. For 1189a, 13 SNPs located on chromosome 6A are predicted to encode missense mutations, with nine deleterious to protein function (SIFT≤0.01) (Table 2). For 2316b, five SNPs located on chromosome 6D are predicted to encode missense mutations, three of which are deleterious (SIFT≤0.02) (Table 3). These deleterious SNPs were prioritized as gene candidates according to ΔSNP indices. Results for 1189a and 2316b converge upon *NAM-A1* (Table 2) and *NAM-D1* (Table 3) (ΔSNP index=1), homoeologues of known senescence regulator *NAM-B1* (Uauy *et al.*, 2006*a*, *b*)

**Fig. 5.**
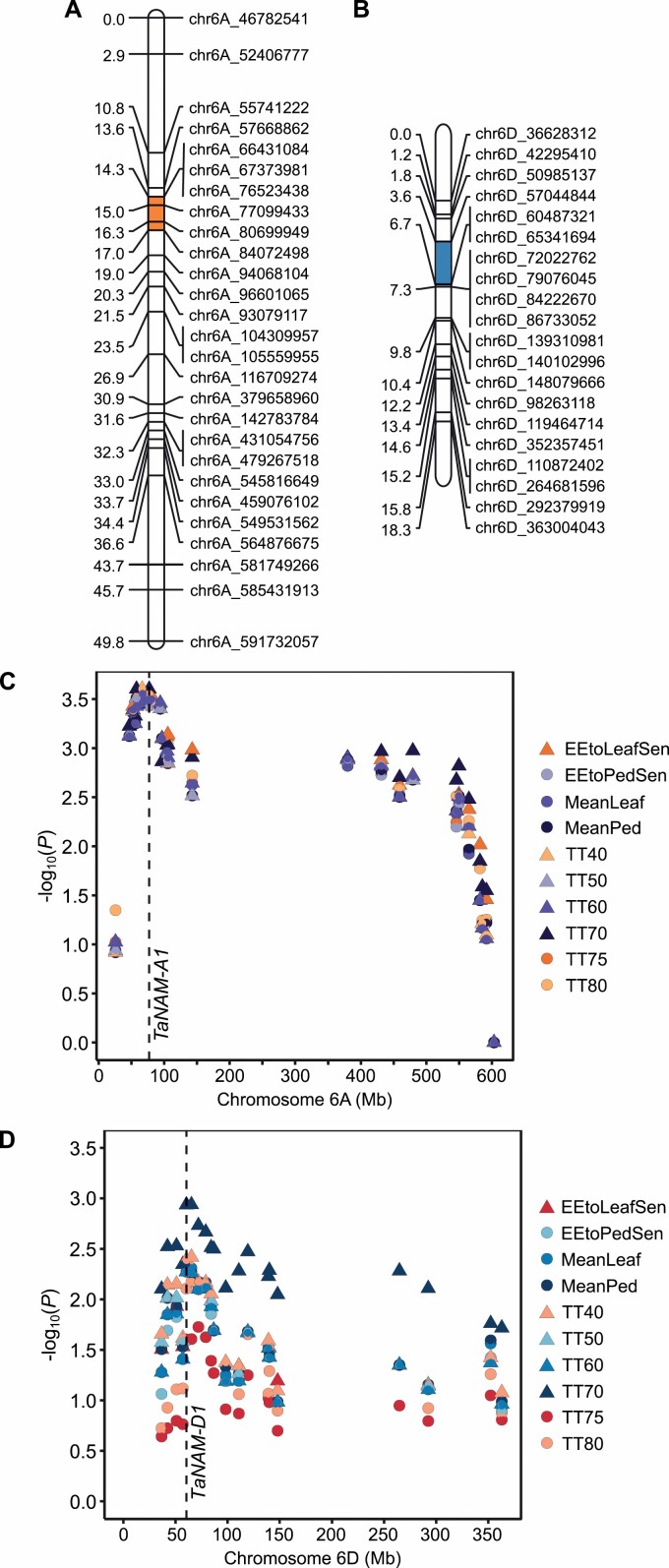
Genetic mapping of *NAM-1* homoeologues as identified by association analysis. (A, B) KASP markers were developed for validation of positions enriched in staygreen bulks, F_4_ RILs genotyped, and genetic maps constructed for regions on chromosome 6A for 1189a (A) and 6D for 2316b (B). (C, D) Single marker association tests were performed using mapped SNPs and senescence phenotypes of Paragon × 1189a (C) and Paragon × 2316b (D) F_4_ RILs (*n*=75) (2018, two replicates). Marker associations increase with proximity to *NAM-A1* and *NAM-D1*, confirming candidate likelihood. Phenotypic abbreviations: EEtoLeafSen and EEtoPedSen, time from ear emergence to terminal flag leaf and peduncle senescence (day °C); MeanLeaf and MeanPed, overall mean flag leaf and peduncle senescence score; TT40, TT50 … TT80, time to flag leaf senescence scores of 40, 50 … 80 (day °C). Highlighted regions indicate ΔSNP index=1 and include SNPs within *NAM-A1* (chr6A_77099433) and *NAM-D1* (chr6D_60487321). Markers named according to RefSeq v1.0 (IWGSC *et al.*, 2018). Marker distances calculated in MapDisto v2.0 (Heffelfinger *et al.*, 2017). Maps constructed using MapChart (Voorrips, 2002).

To validate these results, we developed KASP markers for SNPs located on chromosomes 6A (1189a) and 6D (2316b) (Supplementary Tables S1, S2). Genotyping of F_4_ RIL populations facilitated genetic map construction utilizing all available RILs (Fig. 5A, B). Single marker association analysis reported increasing phenotypic associations with genetic proximity to *NAM-A1* (−log_10_*P*≥3.5) of 1189a (Fig. 5C) and *NAM-D1*, (−log_10_*P*≥2.35) of 2316b (Fig. 5D) (Holm corrected for multiple testing), with size of association reflecting subtlety of senescence phenotype (Fig. 1). Here, we mapped the 1189a interval to a 16.7 Mb region on chromosome 6A and the 2316b interval to a 4.8 Mb region on chromosome 6D, containing 142 and 56 high confidence genes, respectively (Supplementary Dataset S1; IWGSC *et al.*, 2018).

### 1189a and 2316b represent novel sources of *NAM-1* allelic variation

**Fig. 6.**
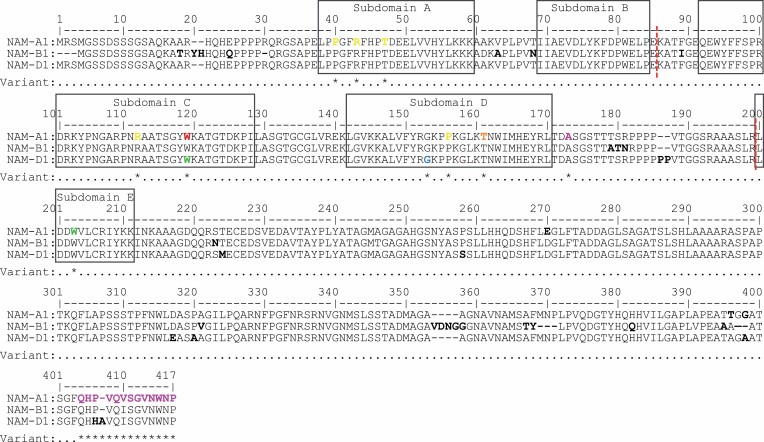
Sequence alignment of wheat NAM-1 homoeologous proteins. Boxes denote NAC subdomains (Ooka *et al.*, 2003). Bold black lettering indicates homoeologous variation, and red dashed lines exon-junctions. Asterisks and bold coloured lettering indicate known variants, including AA substitutions identified for 1189a in orange (G159E, NAM-A1), 2316b in blue (T151, NAM-D1), natural NAM-A1 variants in pink (Cormier *et al.*, 2015), EMS-induced NAM-A1 missense mutations in yellow (Harrington *et al.*, 2019a), EMS-induced knockout mutations in green (Avni *et al.*, 2014) and red (Pearce *et al.*, 2014). In *Triticum aestivum NAM-B1* is largely non-functional due to a +1 bp frameshift mutation, or complete deletion (Uauy *et al.*, 2006b; Hagenblad *et al.*, 2012; Alhabbar *et al.*, 2018*a*, *b*). Accession numbers are, NAM-A1, TraesCS6A02G108300.1; NAM-B1, UniProtKB/Swiss-Prot: A0SPJ4.1 (*Triticum turgidum* ssp*. dicoccoides*); NAM-D1, TraesCS6D02G096300.1.

SNPs identified in *NAM-1* homoeologues encode missense mutations (Tables 2, 3). Isoleucine replaces threonine at amino acid (AA) position 159 of NAM-A1 for 1189a (T159I). Glycine replaces glutamate at AA 151 of NAM-D1 for 2316b (G151E) (Fig. 6). NAC transcription factors operate as heterodimers and homodimers, which ensures stable-DNA binding (Olsen *et al.*, 2005). The G151E and T159I AA substitutions are located within subdomain D of the NAC transcription factor domain at highly conserved positions known to be involved in DNA binding (Ooka *et al.*, 2003; Ernst *et al.*, 2004; Welner *et al.*, 2012; Harrington *et al.*, 2019c). Examination of the NAC domain crystal structure shows that variants mirror one another, with the T159I AA variant located one residue after the β4 structure, and G151E AA variant located one residue before the β5 structure of the antiparallel β-sheet secondary structure (Ernst *et al.*, 2004). The substitution of positively charged threonine for hydrophobic isoleucine, or glycine for a large, positively charged glutamate may affect NAM protein function by altering protein dimerization, as demonstrated for alternative EMS-induced mutations in *NAM-A1* by Harrington *et al.* (2019c) (Fig. 6).

### Differential inheritance of 1189a and 2316b staygreen traits

**Fig. 7.**
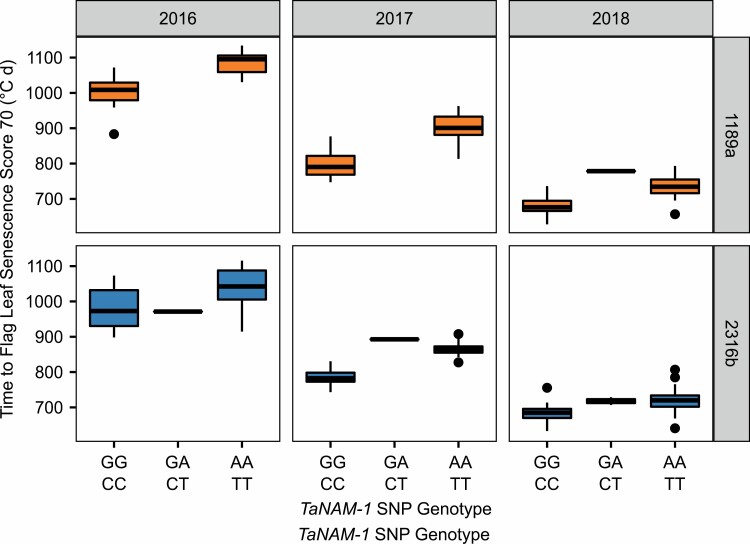
Phenotype×genotype plots illustrate staygreen traits are dominantly inherited. Boxplots displaying TT70 values against *NAM-1* genotype of Paragon × 1189a (orange, top) and Paragon × 2316b (blue, bottom) F_4_ RILs in 2016–2018 (left to right). G/C cv. Paragon allele, A/T mutant allele. Phenotypic differences between contrasting homozygotes are significant (*P*<0.001). Heterozygotes display intermediate phenotypes (*P*>0.05). Mean phenotypic value, *n*=1–3. Black circle, genotypic mean ±SD. Differing numbers of RILs, including heterozygotes, grown each year: 2016, *n*=36; 2017, *n*=43; 2018, *n*>75.

RIL populations were developed by SSD in the glasshouse with segregation of senescence phenotypes unobserved in earlier generations. Phenotype by genotype plots constructed for Paragon × 1189a and Paragon × 2316b F_4_ RILs homozygous for alternative *NAM-1* alleles form distinct groups (*P*<0.0001) (Fig. 7; Supplementary Fig. S8). Assessment of RILs heterozygous for the *NAM-A1* mutation reveal the allele is dominantly inherited, as RILs resemble those homozygous for the mutation (*P*>0.3), not the cv. Paragon allele (*P*<0.01) (Fig. 7).

Inheritance of the 2316b *NAM-D1* mutation is more complex. In 2016 and 2018, senescence phenotypes of heterozygous Paragon × 2316b F_4_ RILs were indistinct from RILs homozygous for either *NAM-D1* allele (2016, *P*>0.7; 2018, *P*>0.28). In 2017 the *NAM-D1* mutation appears dominantly inherited, as senescence of RILs heterozygous and homozygous for the *NAM-D1* mutation was similarly delayed compared with RILs homozygous for the cv. Paragon allele (*P*<0.01) (Fig. 7). Together, this suggests the *NAM-D1* mutant allele is semi-dominant.

Validation of differential modes of inheritance for *NAM-A1* and *NAM-D1* mutations arose from phenotyping heterozygous Ppd × staygreen F_3_ RILs grown in Cambridgeshire and Norwich. Senescence profiles of homozygous RILs contrasting for *NAM-A1* alleles were significantly different (*P*<0.01), with heterozygotes resembling RILs homozygous for the mutation (*P*>0.66) (Supplementary Fig. S9). Penetrance of the *NAM-D1* mutant allele is environmentally dependent. Differences in senescence phenotypes of homozygous RILs contrasting for *NAM-D1* alleles were significant in Cambridgeshire (*P*<0.05) but not in Norwich (*P*>0.5) (Supplementary Fig. S9). *NAM-D1* heterozygotes more closely resemble RILs homozygous for the mutant allele (*P*<0.6) compared with the cv. Paragon allele (*P*>0.9), supporting semi-dominant inheritance of the *NAM-D1* mutation (Supplementary Fig. S9).

## Discussion

### Two novel wheat senescence mutants that extend grain fill duration

Grain filling experiments confirmed the hypothesized positive relationship between staygreen traits and grain fill duration (Wiegand and Cuellar, 1981; Gelang *et al.*, 2000). Grain fill extensions reported for lines 1189a and 2316b mirror observed delays in onset of senescence (Fig. 2; Supplementary Figs S4, S5). Differences in grain fill between staygreen lines and cv. Paragon occur towards the end of the rapid grain filling phase, which Neghliz *et al.* (2016) estimates to occur 39 daa for *Triticum aestivum* cv. Recital when grain moisture content reaches ~45%. Photosynthesis terminates halfway through this rapid phase, whereupon translocation of stored reserves and remobilization of fructose and sucrose occur (Takahashi *et al.*, 1993; Takahashi *et al.*, 2001). Alongside the significant differences in grain moisture content recorded (Supplementary Table S3), we propose a potential extension in the rapid grain filling phase of ~5 d for 1189a and 2316b. The additional grain fill extension observed for 1189a likely relates to differences in the final lag phase of grain fill (Takahashi *et al.*, 2001) as unlike 2316b, grain moisture content was significantly greater compared with cv. Paragon on 23 July in both years (*P*<0.001) (Supplementary Table S3). Delayed grain maturation of 1189a may disrupt depletion of stem reserves and deposition of triticin, glutenin, and gliadin storage proteins occurring during this final phase (Takahashi *et al.*, 2001; Triboï *et al.*, 2003), potentially reducing grain quality and requires further investigation.

Although the pattern of grain moisture decline for our staygreens is environmentally stable, dry grain weight accumulation is under greater environmental influence (Fig. 2; Supplementary Fig. S5). In 2018, the grain fill extension reported for 1189a was associated with increasing final grain weight (*P*<0.001), but the same trend was not evident for 2316b (*P*>0.05) (Supplementary Table 1). In 2018, grain fill was curtailed by ~4–5 d for all lines compared with 2017, with temperatures exceeding the 12–22 °C range considered optimal (Dias and Lidon, 2009; Farooq *et al.*, 2011; Supplementary Fig. S2). Therefore, the greater final grain weight of 1189a reveals an association between staygreen traits and stress tolerance, as reviewed by Gregersen *et al.* (2013) and Thomas and Ougham (2014).

Final grain weight and grain filling rate are significantly correlated (Dias and Lidon, 2009), with slower rates reducing remobilization efficiency (Xie *et al.*, 2015). Grain filling rate of these staygreens may be affected, illustrated by the shallower gradients between time points when grain moisture contents are significantly different, and are potentially slowest for 1189a (Fig. 2; Supplementary Fig. S4). The later termination of photosynthesis for 1189a, and greater availability of photosynthates, may counteract the reduction in stem reserve remobilization under stress, explaining the perhaps contradictory final grain weight increase in 2018. Evidence of additional photosynthates sustaining grain fill of staygreens are the greater dry grain weights recorded for 1189a from 42 daa, and 2316b from 37 daa (*P*<0.05) (Supplementary Table S3). Grain filling experiments by Borrill *et al.* (2015) support this, with flag leaves of *NAM-*RNAi lines producing 2079 mg more glucose per plant compared with controls. For 2316b, these earlier increases did not improve final grain weight suggesting remobilization efficiency may be impaired like 1189a, with earlier termination of senescence unable to compensate. Alternatively, any final grain weight improvement associated with 2316b may be diluted due to background mutations.

### Evaluating mapping by exome capture

Adopting an exome capture-enabled BSA we mapped two staygreen traits to likely causative mutations in *NAM-1* homoeologues. In wheat, the use of exome capture-enabled BSA has typically been limited to mapping of qualitative traits, including yellow rust resistance (Ta*Yr6*; Gardiner *et al.*, 2016), plant height (Ta*RhtB1*; Mo *et al.*, 2018) and a dominant chlorosis phenotype (*YES-1* locus; Harrington *et al.*, 2019*b*). However, in combination with the recent mapping of a novel Ta*MKK-3* allele conferring pre-harvest sprouting resistance (Martinez *et al.*, 2020), our results illustrate BSA can be used to map quantitative traits.

Whilst parental senescence phenotypes were distinct (Table 1), variation between RILs was often limited (Fig. 3), preventing senescence being scored as a binary trait as per Harrington *et al.* (2019*b*). Instead, RILs were classified relative to parents whereupon within- and between-year comparisons of multiple senescence metrics addressed variable trait expressivity and stability. Subsequent calculation and reporting of ΔSNP index=1 indicate high accuracy of RIL selection, contributing to the success of the BSA approach. Conversely, if RILs for bulk inclusion were decided based solely on 2016 data, accuracy would have been reduced, as retrospective analysis revealed 25–30% of RILs were classified differently upon evaluation of 2017 data.

BSA mapped 1189a and 2316b staygreen traits to 16.7 and 8.2 Mb genetic regions (Tables 2, 3), with use of additional recombinants halving this to 4.8 Mb for 2316 (Fig. 5). Conversely, for 1189a no additional recombinants were identified despite similar size of Paragon × staygreen F_4_ RIL populations. Indeed, markers developed for mutations within the identified 6A region for 1189a were in complete linkage, located in a reportedly highly conserved haplotype block (Brinton *et al.*, 2020), thereby preventing dissection of the locus in the absence of hundreds of additional RILs. More widely, the size of regions we identified compared favourably with studies applying similar methods, which range from 1.9 to 32.9 Mb (Mo *et al.*, 2018; Harrington *et al.*, 2019*b*; Martinez *et al.*, 2020).

### *NAM-A1* and *NAM-D1* as gene candidates

Regions identified by BSA for 1189a and 2316b encode mutations in *NAM-1* homoeologues. *NAM-B1* is a known senescence regulator, previously identified in *Triticum turgidum* ssp*. dicoccoides* during dissection of locus *GPC-1* (Uauy *et al.*, 2006*a*, *b*). In most hexaploid wheats *NAM-B1* is non-functional due to a +1 bp frameshift-encoding insertion or its complete deletion (Uauy *et al.*, 2006b; Hagenblad *et al.*, 2012; Lundström *et al.*, 2017). The role of *NAM-A1* and *NAM-D1* homoeologues in senescence was confirmed by Avni *et al.* (2014), with natural variation limited to A and B homoeologues with no D variants reported. Of the *NAM-A1* alleles detected, one encodes an alanine to valine AA substitution between subdomains D and E (A171V), the other a frameshift-induced truncation mutation (Fig. 6) (Cormier *et al.*, 2015).

SNPs within *NAM-1* homoeologues of 1189a and 2316b are considered deleterious to protein function (SIFT=0) (Tables 2, 3) and are completely associated with senescence phenotypes (Fig. 5). Supporting the proposal of these mutations as causative is a reverse genetic study concerning *gpc-1* (*NAM-1*) TILLING mutants by Avni *et al.* (2014) (Fig. 6). A delay in onset of senescence of ~6 d and ~3 d was reported for the *Triticum aestivum* cv. Express *gpc-a1* W196* truncation mutant and *gpc-d1* W114* knockout mutant, respectively (Avni *et al.*, 2014), matching the phenotypes recorded for 1189a and 2316b (Table 1). Likewise, senescence of *gpc-1* mutants and 1189a and 2316b progresses in parallel, terminating 6–10 d and 5 d later compared with cv. Express, respectively (Avni *et al.*, 2014; Supplementary Fig. S3).

Within a protein context, mutations within *NAM-A1* and *NAM-D1* are self-validating, encoding AA substitutions within subdomain D of the NAC domain that encompasses a DNA binding region (Fig. 6) (Ooka *et al.*, 2003; Welner *et al.*, 2012). Mutations in subdomain D can alter NAM protein functionality, with the affected G151 and T159 residues conserved in over 65% of NAC transcription factor encoding genes (Ooka *et al.*, 2003; Puranik *et al.*, 2012; Welner *et al.*, 2012; Fan *et al.*, 2014). In *Triticum turgidum* TILLING mutants, a P154L mutation in NAC subdomain D of NAM-A1 disrupted protein dimerization in the absence of a senescence phenotype (Harrington *et al.*, 2019c), whilst a G133D mutation in subdomain D of NAM-A2, the *NAM-A1* paralogue, significantly delayed peduncle senescence (Borrill *et al.*, 2019). Similarly, *NAM-1* homologues encoding subdomain D allelic variants in *NAM-G1* of *Triticum timopheevi* and *NAM-1* of *Hordeum vulgare* are associated with reducing grain protein content (*P*<0.05), illustrating loss of function (Jamar *et al.*, 2010; Hu *et al.*, 2013).

Welner *et al.* (2012) proposed that one NAC monomer initially sub-optimally binds DNA with the other scanning and searching for a binding site. Changes in charge or polarity introduced by G151E and T159I mutations may disrupt initial DNA-binding to prevent dimerization, with NAM-1 homoeologues affected similarly due to structural palindromicity of residues (Ernst *et al.*, 2004; Welner *et al.*, 2012). Performance of yeast-2-hybrid and cell death assays for mutated NAM-1 proteins would test for altered binding activity, as performed by Harrington *et al.* (2019c), to identify residues critical to NAM-A1 protein function. The extremity of the 1189a staygreen phenotype compared with 2316b likely reflects homoeologous dominance of *NAM-A1* over *NAM-D1* and not mutation type, although involvement of linked mutations within mapped intervals not captured by exome sequencing, including promoter variants, cannot be disregarded. However, the delays in onset of senescence recorded for 1189a and 2316b match those reported by Avni *et al.* (2014) in a study of *T. aestivum* cv. Express *gpc-1* mutants, thereby providing independent supporting evidence.

### Grain fill phenotypes of 1189a and 2316b compare favourably with *NAM-1* variants

When characterizing *GPC-B1* (*NAM-B1*) Uauy *et al.* (2006*a*) reported an association between a non-functional allele and longer grain filling period. The results of Avni *et al.* (2014) validate the association between variation and grain fill extension observed for 1189a (*NAM-A1*) and 2316b (*NAM-*D1) mutants. From 42 daa spike moisture content of *gpc-1* mutants was greater when compared with parental controls (*P*<0.05) and remained so until 49 daa and 57 daa for the *gpc-d1* and *gpc-a1* mutant, respectively, matching the pattern of grain moisture loss recorded for 1189a and 2316b (Fig. 2; Supplementary Fig. S4). *NAM-A1* variants are common in Australian wheat cultivars with variation characteristic of mid to mid-late maturity types (Alhabbar *et al.*, 2018*a*), just as observed for 1189a (Fig. 2).

Contrary to the hypotheses of Wiegand and Cuellar (1981), Gelang *et al.* (2000), and Bogard *et al.* (2011) delayed senescence and grain fill extension, as associated with *NAM-1* variation, may not improve final grain weight. During grain fill, dry grain weights recorded for 1189a and 2316b were greater (*P*<0.05) (Supplementary Table S3), contradicting the lower weights recorded for *NAM-1* RNAi lines (*P*<0.05) (Borrill *et al.*, 2015) and similar dry spike weights recorded for *gpc-1* mutants (*P*>0.05) (Avni *et al.*, 2014). Differences in final grain weight between *NAM-1* RNAi, 2316b, and *gpc-1* mutant lines and controls were not significant (*P*>0.05) (Avni *et al.*, 2014; Borrill *et al.*, 2015), but grain weight of 1189a was greater in 2018 (*P*<0.001) (Supplementary Table S3).

The influence of *NAM-1* variation on final grain weight depends on genetic background and environment. Differences in TGW of isogenic lines carrying a non-functional *GPC-B1* copy were inconsistent and both higher and lower (*P*<0.05) (Uauy *et al.*, 2006*a*), and likewise for cultivars carrying *NAM-A1* variants (Cormier *et al.*, 2015; Alhabbar *et al.*, 2018*a*). Borrill *et al.* (2015) hypothesized similarity in TGW of *NAM-1* RNAi lines and controls (*P*=0.25) was due to inadequate starch synthase activity. Instead of contributing to grain filling activities, Borrill *et al.* (2015) demonstrated the additional sucrose synthesized by *NAM-1* RNAi lines was retained as stem fructan within the internodes. Improved stem fructan remobilization is associated with TGW improvement (Zhang *et al.*, 2015), with fructan providing a source of water-soluble carbohydrates for sustainment of grain filling (Fischer, 2011; Borrill *et al.*, 2015). Greater final grain weight of 1189a in 2018 and recorded during grain filling for 2316b (*P*<0.05) (Supplementary Table S3) demonstrate that the novel *NAM-1* alleles we identified could increase TGW. Therefore, combining these *NAM-1* alleles with a mutation in gene *1-FEH-w3* regulating stem fructan remobilization (Zhang *et al.*, 2015) could overcome problems associated with fructan retention (Borrill *et al.*, 2015) and consistently improve TGW.

### Dominance of *NAM* genes in regulation of wheat senescence

Genetic mapping of 1189a and 2316b converged upon homoeologous copies of known senescence regulator *NAM-B1* (Uauy *et al.*, 2006b). Similarly, a forward genetic screen of a *Triticum turgidum* cv. Kronos TILLING population to identify senescence mutants converged upon *NAM-A1* mutations (Harrington *et al.*, 2019*b*). The differential onset of senescence observed for 1189a and 2316b (Table 1) reflects the reported dominance of *NAM-1* homoeologues (Avni *et al.*, 2014), with our results the first forward genetic screen identifying *NAM-D1*.

*NAM-1* is a positive regulator of senescence, with expression up-regulated following anthesis (Uauy *et al.*, 2006b), and is associated with transcriptional reprogramming. At 12 daa RNA-seq studies of *gpc-1* (*NAM-1*) mutants identified ≥691 differentially expressed genes, with protein catabolism and stress response genes up-regulated, and photosynthetic and housekeeping genes down-regulated (Cantu *et al.*, 2011; Pearce *et al.*, 2014; Borrill *et al.*, 2019). The role of *NAM* genes in senescence regulation is complicated by the *NAM-1* paralogue *NAM-2*. RNA-seq studies of tetraploid wheat *gpc-1* and *gpc-2* mutants identified *NAM-1* as dominant over *NAM-2*, with expression associated with 64% of senescence-regulated genes compared with 37%, respectively (Pearce *et al.*, 2014). Phenotypic characterization of *NAM-B2* mutants confirmed its role in senescence regulation, but phenotypic differences were only significant when combined with mutations in *NAM-A1* or *NAM-A2* (*P*<0.05) (Pearce *et al.*, 2014; Borrill *et al.*, 2019). These findings illustrate the complexity of senescence and the problems associated with identification of genetic regulators of senescence using forward genetic techniques.

Mutations identified in *NAM-1* homoeologues were penetrative and dominant (Fig. 7), with their detection relatively unconfounded by homoeologues as cv. Paragon encodes a non-functional copy of *NAM-B1*. Due to the apparent dominance of *NAM-1*, it is possible mutations in genes acting downstream result in more subtle phenotypes. Inconsistencies between leaf and peduncle phenotypes (Borrill *et al.*, 2019; Harrington *et al.*, 2019*b*, *c*), spatial variation or effects of background mutations may prevent identification of other contributing loci. Whilst the original screen of the *Triticum aestivum* cv. Paragon EMS mutant population identified lines 1189a and 2316b to be of interest, delayed and accelerated senescence phenotypes were confirmed for additional lines. Therefore, this resource is worth further exploration to shed further light of the genetic regulation of senescence in wheat. For example, this could include sequencing of *NAM-1* and *NAM-2* homoeologues of staygreen mutants to identify causative mutations due to their known, and overarching, role in senescence regulation (Avni *et al.*, 2014; Pearce *et al.*, 2014; Borrill *et al.*, 2019; Harrington *et al.*, 2019c). Conversely, early senescing mutants may encode gain of function mutations affecting *NAM-1* gene regulatory targets including C2C2-CO like transcription factors, RWD-RK, or GRAS genes identified during transcriptional network modelling (Borrill *et al.*, 2019; Harrington *et al.*, 2019a).

## Conclusions

Here, we confirm the central role of *NAM-1* in the genetic regulation of wheat senescence through identification of novel mutant *NAM-A1* and *NAM-D1* alleles following a forward genetic screen. Both mutations occurred within subdomain D of the NAC domain, highlighting the importance of this subdomain in modulating NAM-1 function. Altered senescence profiles associated with these mutations are independent of heading-date variation and contribute to a grain fill extension and potential increased grain weight, whereby introduction and selection of these novel *NAM-1* alleles could enhance final grain yield.

## Supplementary data

The following supplementary data are available at *JXB* online.

Fig. S1. Development of RIL populations segregating for senescence traits and mapping strategy.

Fig. S2. Daily rainfall and mean daily temperature data.

Fig. S3. Senescence phenotypes for lines 1189a and 2316b (2016 and 2018).

Fig. S4. Delayed senescence is associated with grain fill extension in 2017.

Fig. S5. Senescence phenotypes corresponding to grain filling experiments (2018).

Fig. S6. TT70 scores for Paragon × 1189a F_4_ RILs (2016 to 2018).

Fig. S7. TT70 scores for Paragon × 2316b F_4_ RILs (2016 to 2018).

Fig. S8. Additional phenotype×genotype plots illustrating mode of inheritance.

Fig. S9. Independent confirmation of modes of inheritance for *NAM-1* mutations.

Table S1. KASP primers for 1189a.

Table S2. KASP primers for 2316b.

Table S3. Differences in grain filling parameters, pairwise-comparison (2017 and 2018).

Table S4. Exome capture coverage and identified SNPs.

Dataset S1. RIL senescence classification & bulk selection; Gene lists for mapped intervals according to Chinese Spring on chromosome 6A and 6D of 1189a and 2316b, respectively.

erab368_suppl_Supplementary_Materials_1

erab368_suppl_Supplementary_Materials_2

## Data Availability

Raw exome capture sequencing reads for all samples have been deposited on the European Nucleotide Archive (ENA) (PRJEB40428) at https://www.ebi.ac.uk/ena. All germplasm, including original EMS mutants and associated RIL populations, are available from the Germplasm Resources Unit (GRU) at John Innes Centre. Data supporting the findings of this study are available within the paper and within its supplementary data published online. Additional data can be requested from the corresponding author (EAC) upon request.

## Abbreviations

AA: amino acid
BSA: bulk segregant analysis
daa: days after anthesis
EMS: ethyl methane sulfonate
JIC: John Innes Centre
KASP: kompetitive allele specific PCR
RIL: recombinant inbred line
SSD: single seed descent
TGW: thousand grain weight
TILLING: targeting induced local lesions in genomes
TTxx: thermal time to flag leaf senescence score
