# Supplementary material for: Delaying or delivering: identification of novel NAM-1 alleles that delay senescence to extend wheat grain fill duration
Source: J Exp Bot. 2021 Aug 18;72(22):7710–28. doi: 10.1093/jxb/erab368 (PMC8660559; doi:10.1093/jxb/erab368)
Supplement: erab368_suppl_Supplementary_Materials_1 [file erab368_suppl_supplementary_materials_1.docx]

**Supplementary Material 1**

**
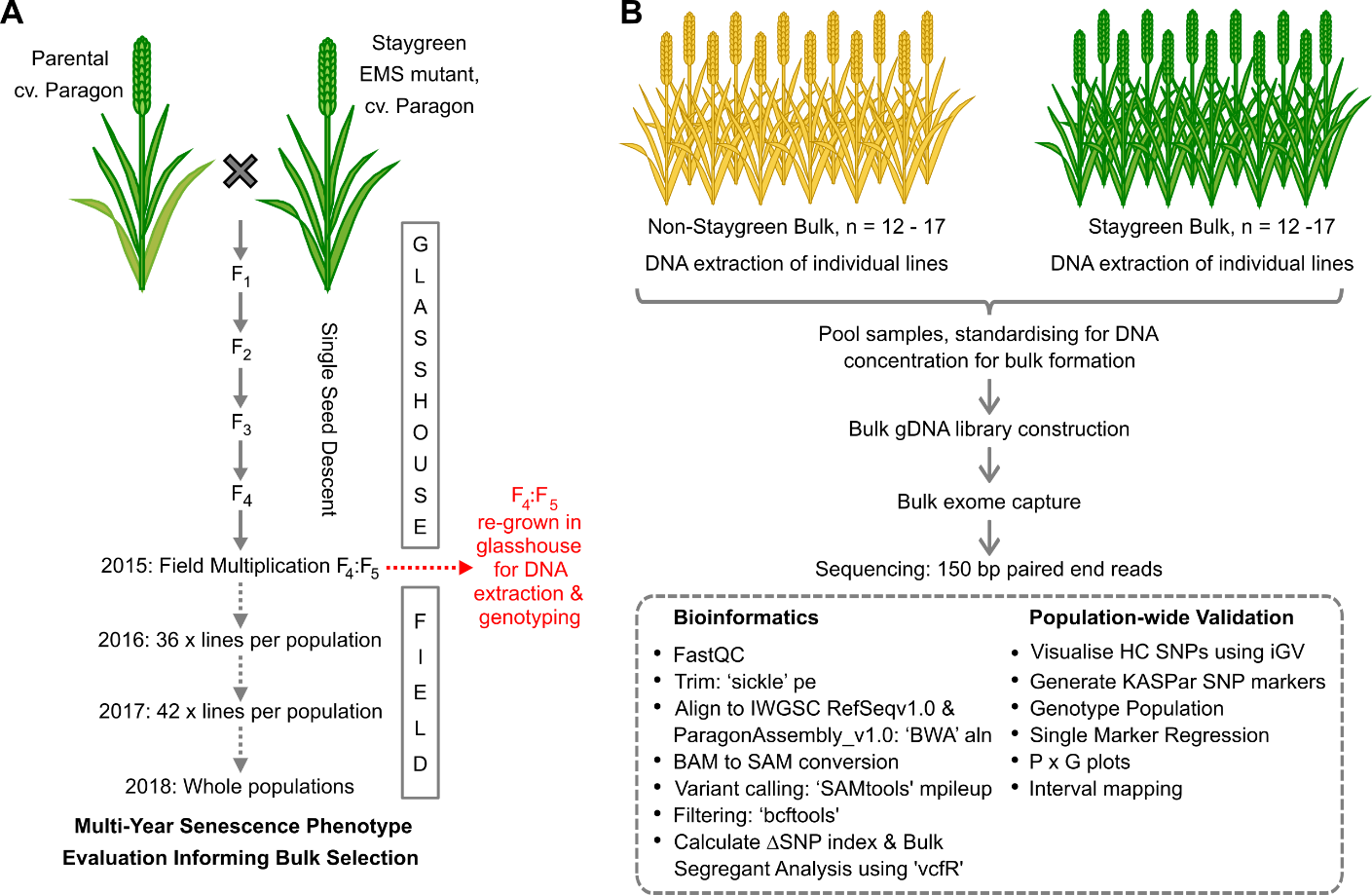
**

**Figure S1.** **Development of RIL populations segregating for senescence traits and mapping strategy.** **(A)** Staygreen mutants were crossed to cv. Paragon and F_4_ RIL population developed through SSD. Differing numbers of RILs underwent phenotypic assessment in the field between 2016-2018. **(B)** Following classification of RILs as ‘staygreen’ or ‘non-staygreen’, DNA of selected RILs was pooled to form contrasting bulks and submitted for exome capture and sequencing alongside parents. Sequencing reads were subject to QC analysis and processing prior to read mapping and variant detection facilitating performance of BSA. KASP markers were developed for SNPs of interest for genotyping of entire RIL populations and marker association analysis performed.

**
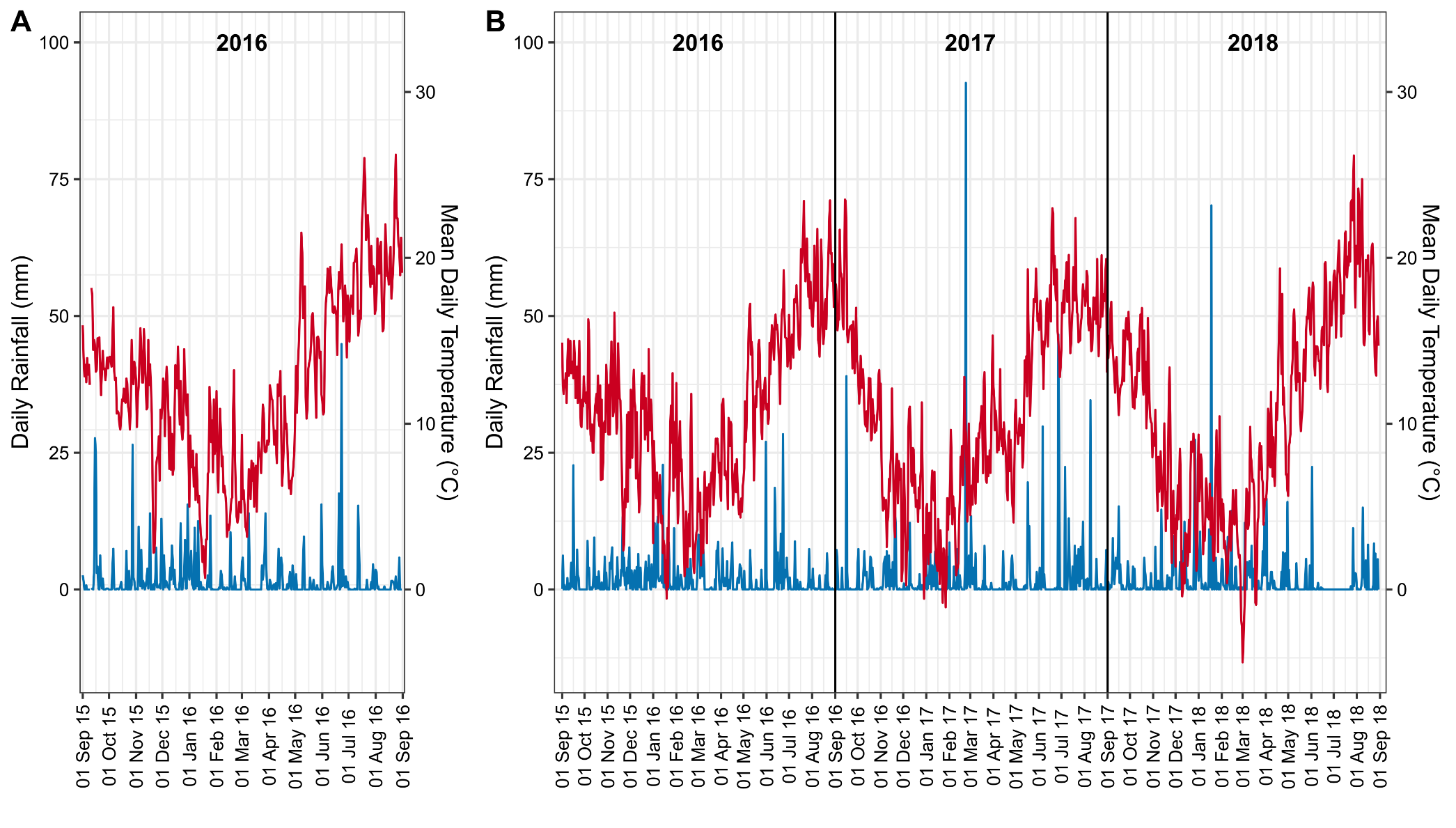
**

**Figure S2:** **Daily rainfall (mm) and mean daily temperature (°C)**. **(A)** Cambridgeshire in 2016. **(B)** Cburch Farm, Bawburgh, Norwich (JIC) from September 2015 to 2018 **(B)**.

**
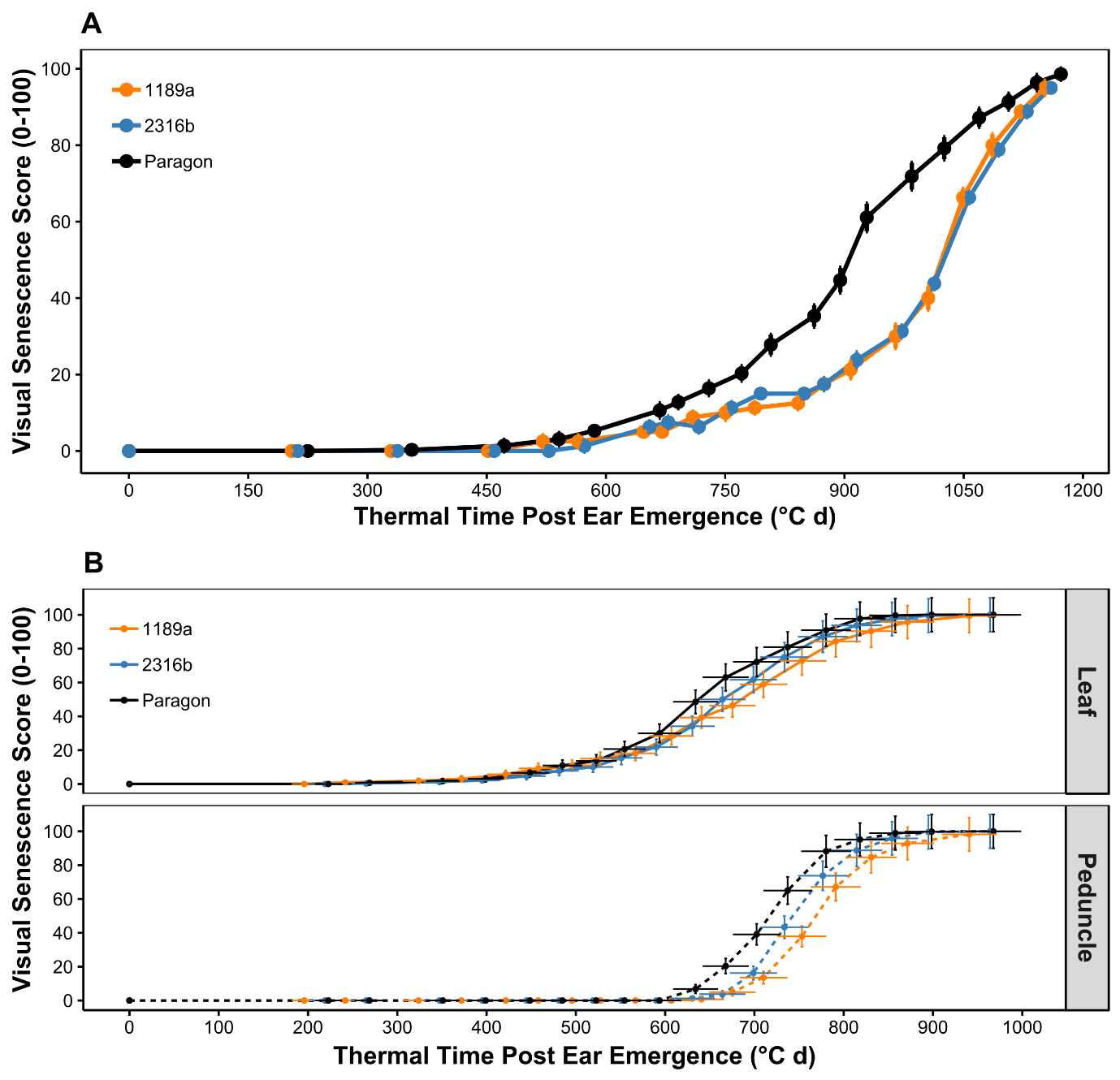
**

**Figure S3.** **A delay in onset of senescence is characteristic of 1189a and 2316b staygreen phenotypes.** Onset of leaf senescence was delayed for both mutants in 2016 **(A)**, alongside peduncle senescence in 2018 **(B)**. Differences in senescence progression were usually significant, P-value < 0.1 (Table 1). Senescence was scored visually using a 0-100 scale every 2-4 times per week from ear emergence (GS55) (07/06/2016 ± 1.4, 31/05/2018 ± 1.1), with scoring dates converted to thermal time (day °C). Mean ± SEM, n ≥ 4. Lines shown, cv. Paragon (black), 1189a (orange), 2316b (blue).

**
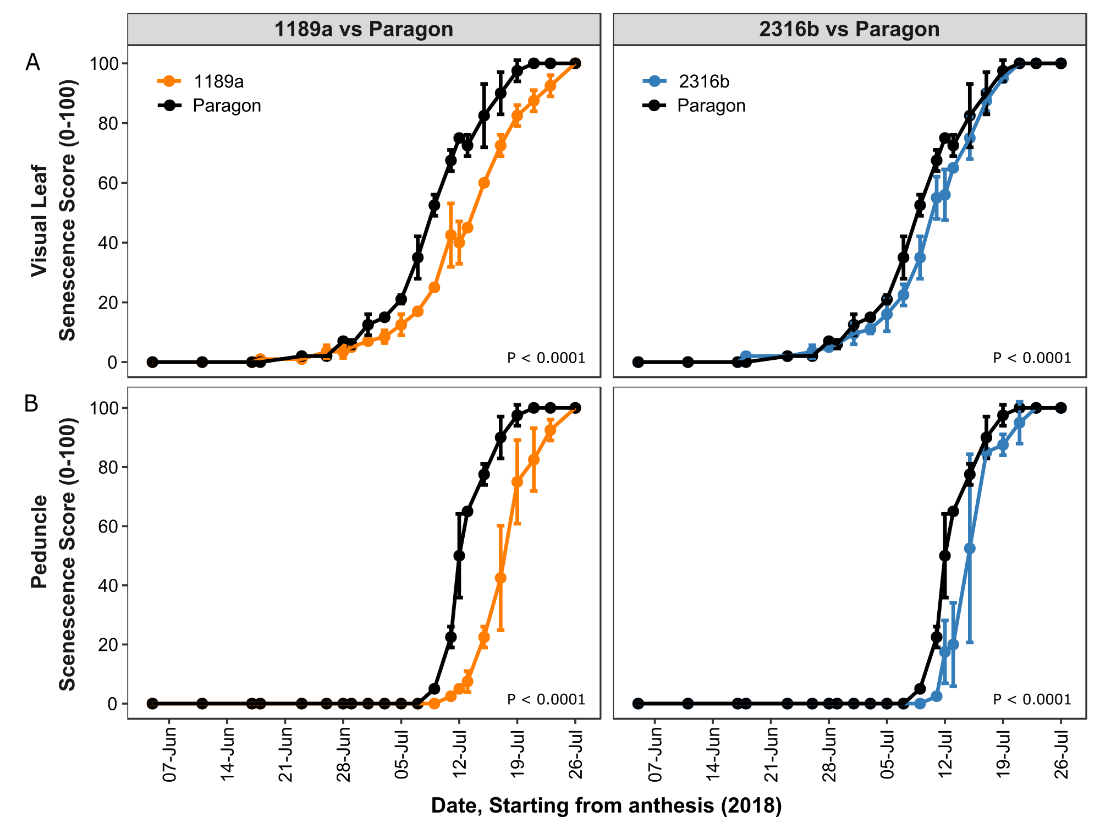
**

**Figure S4. Senescence phenotypes corresponding to lines used in grain filling experiments in 2018.** Progression of flag leaf **(A)** and peduncle **(B)** senescence for lines 1189a (orange, left) and 2316b (blue, right) is significantly delayed compared to cv. Paragon, P-value < 0.0001. Senescence and grain filling phenotypes mirror one another and relate to severity in onset. Senescence scored visually using a 0-100 scale 2-4 times per week from anthesis (05/06/2018). Mean ± SD, n = 2.

**
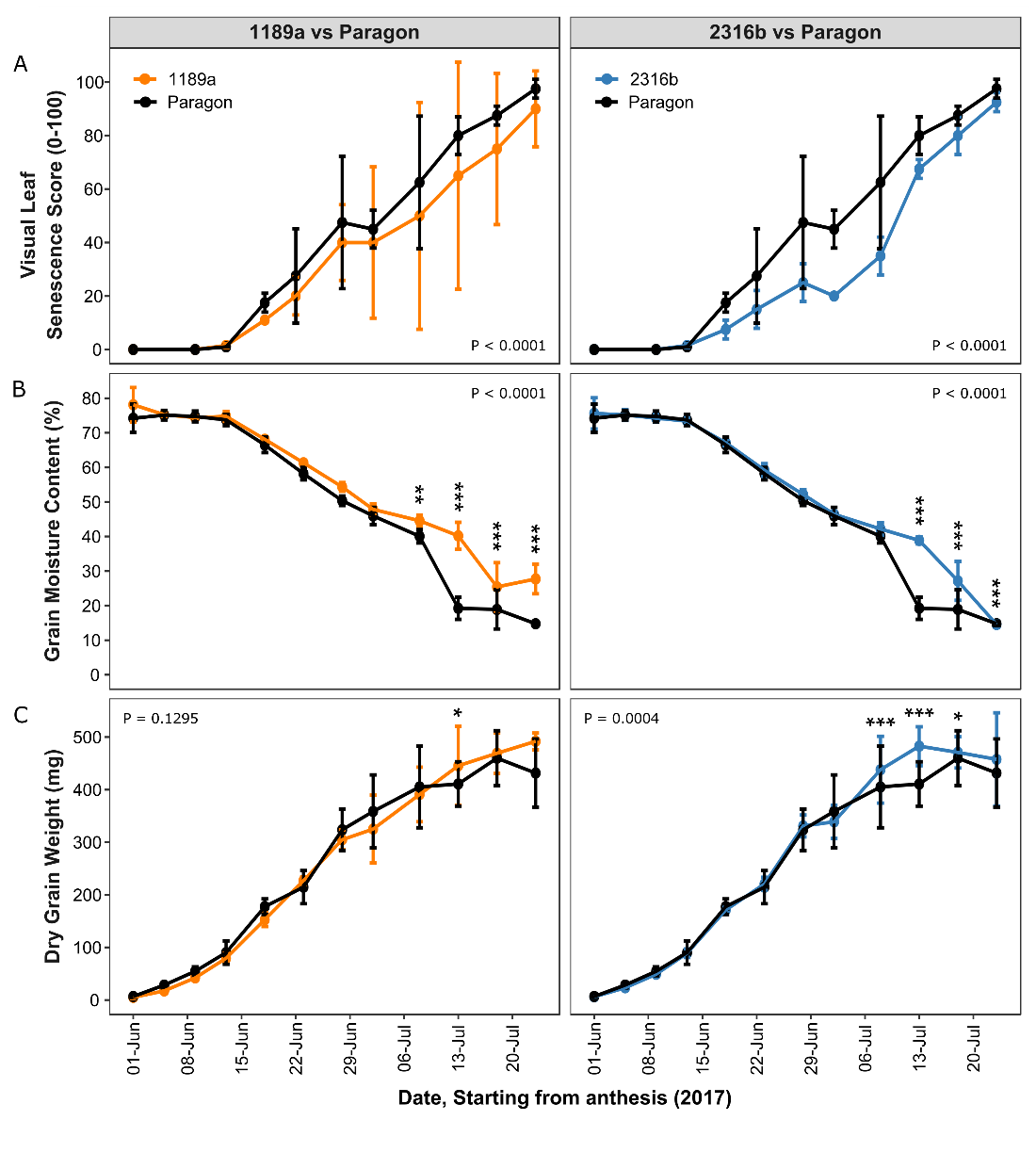
**

**Figure S5. Delayed senescence is associated with grain fill extension.** Results of grain filling experiments conducted for 1189a (orange, left) and 2316b (blue, right) in 2017. 37 daa grain moisture content of mutant lines remained elevated compared to cv. Paragon, P-values < 0.01 **(B)**. Frequency of which significant differences in grain moisture content are recorded reflect differences in senescence duration of 1189a and 2316b compared to cv. Paragon **(A)**. Differences in dry weight accumulation over time were significant between line 2316b and cv. Paragon, P-value < 0.01, but not 1189a, P-value > 0.1 **(C)**, and were unrealised regarding final grain weight. Senescence, grain moisture content (%) and dry grain weight (10 grains in mg) were recorded at 3 to 5-day intervals starting from anthesis (01/06/2017). Mean ± SD, n = 4-5, 1 plot per line. Printed P-values represent overall differences (ANOVA). Pairwise differences indicated at corresponding time points, P-values: * < 0.05, ** < 0.01, *** < 0.001 (Tukey post-hoc test).

**
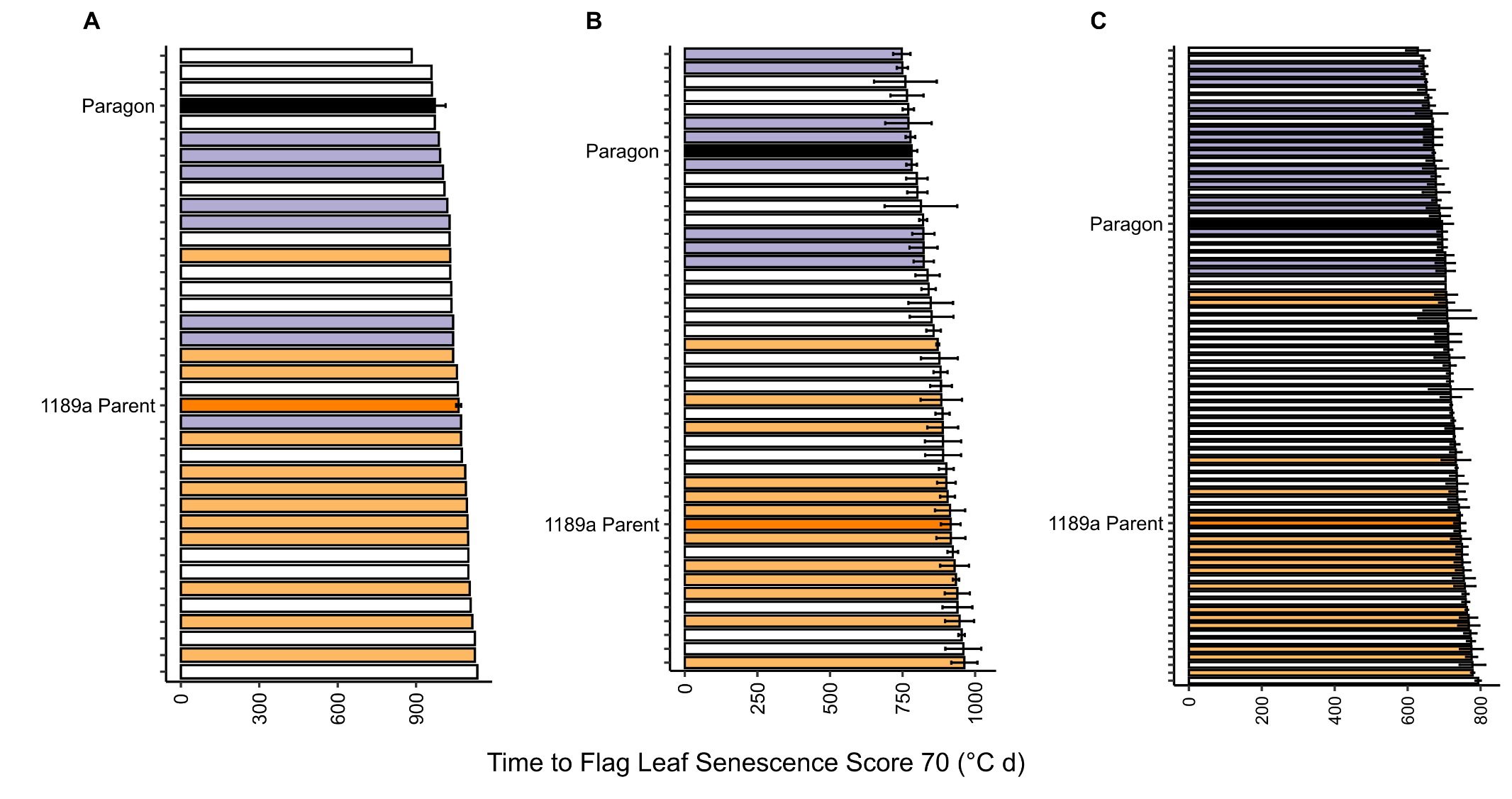
Figure S6. TT70 scores for Paragon x 1189a F_4_ RILs (2016 to 2018).** Senescence metric **‘**Time to visual leaf senescence score 70 (°C d)’ proved reliable in identifying senescence extremes for inclusion in bulks. Mean ± SD for each RIL in **(A)** 2016, n = 1; **(B)** 2017, n = 3; **(C)** 2018, n = 2. Coloured bars represent parents and RILs included in bulks, 1189a (orange), cv. Paragon (black), ‘staygreen’ (light orange, n = 17), ‘non-staygreen’ (light purple, n = 17).

**
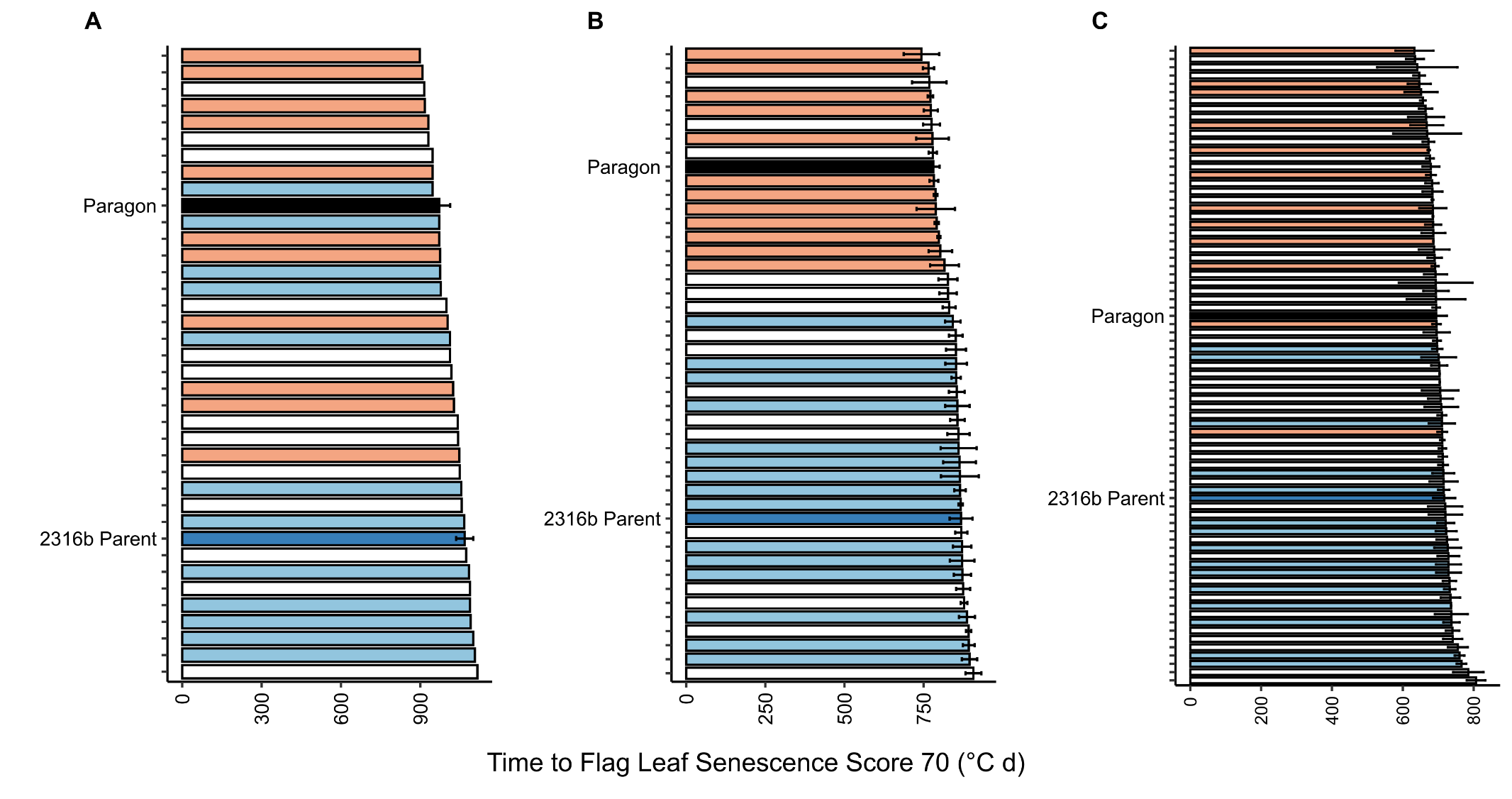
 Figure S7. TT70 scores for Paragon x 2316b F_4_ RILs (2016 to 2018).** Senescence metric **‘**Time to visual leaf senescence score 70 (°C d)’ proved reliable in identifying senescence extremes for inclusion in bulks. Mean ± SD for each RIL in **(A)** 2016, n = 1; **(B)** 2017, n = 3; **(C)** 2018, n = 2. Coloured bars represent parents and RILs included in bulks, 2316b (dark blue), cv. Paragon (black), ‘staygreen’ (light blue, n = 15), ‘non-staygreen’ (red, n = 12).

**
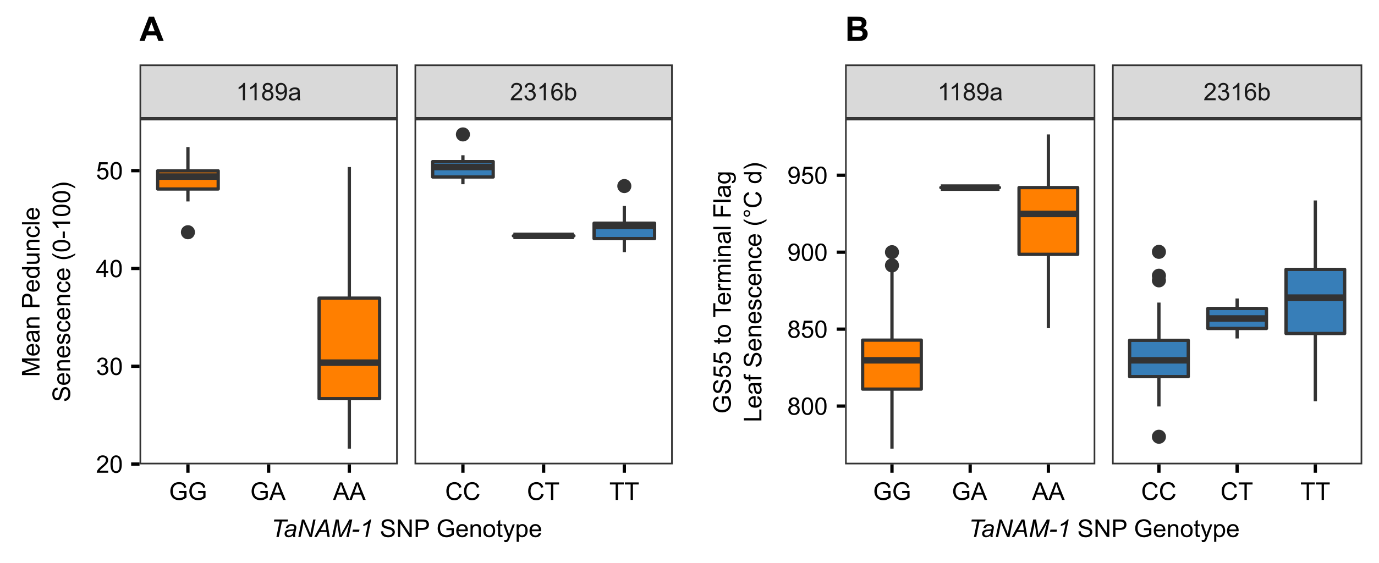
**

**Figure S8. Additional Phenotype x Genotype plots illustrating mode of inheritance.** Boxplots displaying mean peduncle senescence values recorded in 2017 **(A)** and duration of leaf senescence (from GS55) recorded in 2018 **(B)** against *NAM-1* genotype. Paragon x 1189a F_4_ RILs (orange, left), Paragon x 2316b F_4_ RILs (blue, right). G/C cv. Paragon allele, A/T mutant allele. Phenotypic differences between contrasting homozygotes are significant, P-value < 0.001. Heterozygotes display intermediate to staygreen phenotypes. Black circle, genotypic mean ± SD. Differing numbers of RILs, including heterozygotes, grown each year, 2017, n = 43; 2018, n > 75.

**
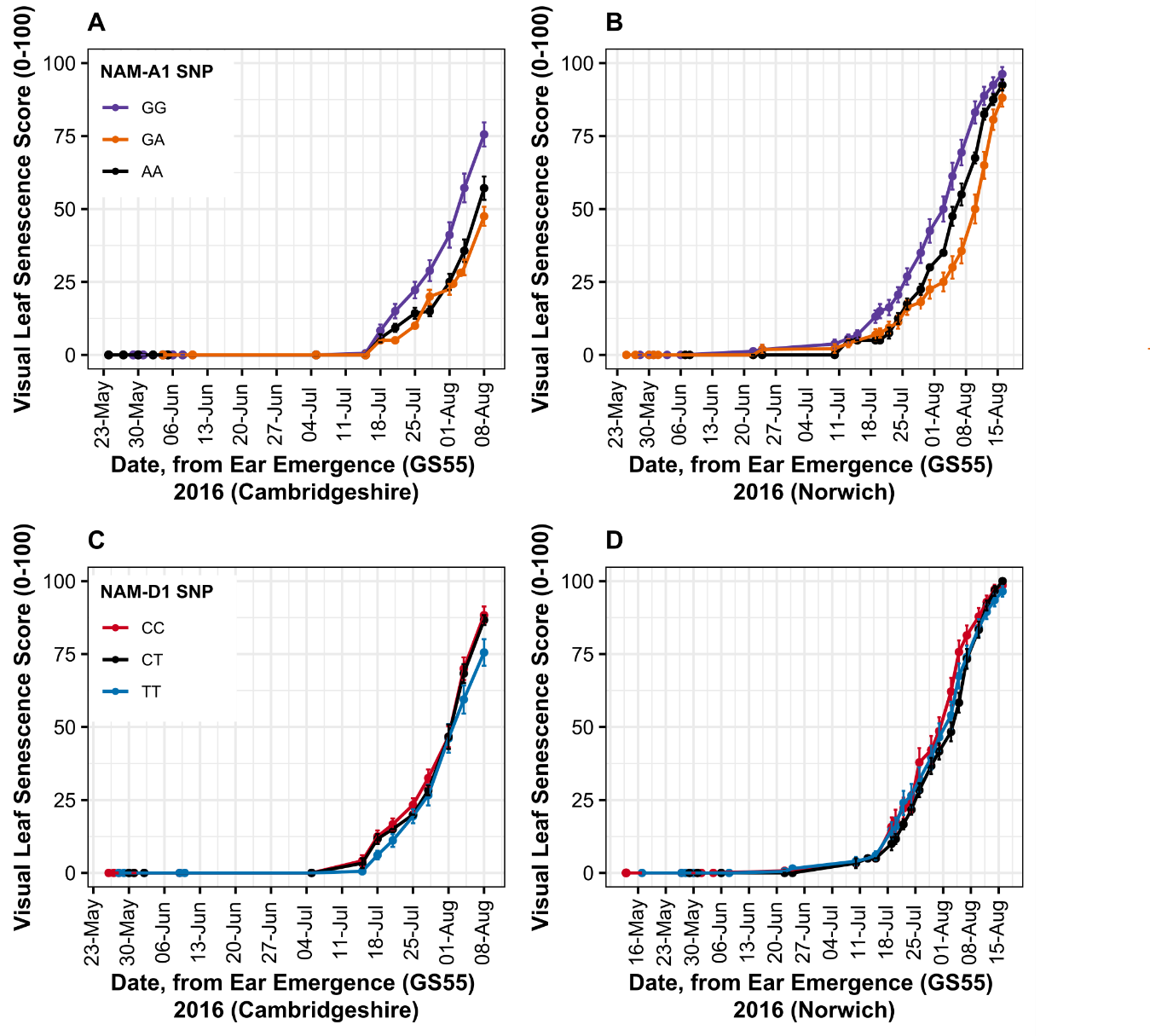
**

**Figure S9. Observation of co-dominant and additive modes inheritance for *NAM-1* mutations.** Senescence profiles of F_3_ RILs heterozygous for *NAM-A1* and *NAM-D1* mutations (from independent crosses) support proposed differential modes of inheritance. Senescence profiles of homozygous RILs contrasting for *NAM-A1* were significantly different, P-value < 0.01, when grown in Cambridgeshire **(A)** and Norwich **(B)**, with heterozygotes resembling RILs homozygous for the *NAM-A1* mutation, P-value > 0.66. Phenotypic penetrance of the *NAM-D1* mutation is subject to greater environmental interaction. Differences in senescence progression of homozygous RILs contrasting for *NAM-D1* were significant when grown in Cambridgeshire, P-value < 0.05 **(C)**, not Norwich, P-value > 0.55 **(D)**. *NAM-D1* heterozygotes resemble RILs homozygous for the *NAM-D1* mutation, P-value > 0.9, and display intermediate to staygreen phenotype, supporting an additive mode of inheritance. Senescence was scored visually using a 0-100 scale every 2-4 times per week from ear emergence (GS55).

**Table S1.** *Differences in grain filling parameters correspond to severity in onset of senescence.*

Mean [CI_95%_], results of pairwise Tukey post-hoc tests following ANOVA. P-values, * < 0.05, ** < 0.01, *** < 0.001. Corresponding graphs, Supplemental Figure S3 (2017) and Figure 3 (2018). Grain sampling started at anthesis, which occurred on 01/06/2017 and 05/06/2018.

|  | **Grain Filling**  **Component** | **2017 (dd/mm)**  **& days after anthesis (daa)** | | | | **2018 (dd/mm)**  **& days after anthesis (daa)** | | | | |
| --- | --- | --- | --- | --- | --- | --- | --- | --- | --- | --- |
| **Genotype** |  | **08/07**  **37 daa** | **13/07**  **42 daa** | **18/07**  **47 daa** | **23/07**  **52 daa** | **12/07**  **37 daa** | **17/07**  **42 daa** | **20/07**  **45 daa** | **23/07**  **48 daa** |  |
| **Paragon** | **Moisture**  **Content (%)** | **40.1**  **[37.7, 42.5]** | **19.2**  **[16.8, 21.6]** | **18.9**  **[16.5, 21.3]** | **14.8**  **[12.4, 17.2]** | **39.0**  **[37.5, 40.6]** | **16.2**  **[14.7, 17.8]** | **11.8**  **[10.2, 13.3]** | **8.2**  **[6.6, 9.7]** |  |
|  | **Weight**  **(10 grains in mg)** | **391**  **[370, 412]** | **420**  **[399, 441]** | **459**  **[438, 480]** | **438**  **[417, 459]** | **482**  **[436, 501]** | **423**  **[404, 442]** | **437**  **[418, 456]** | **443**  **[424, 462]** |  |
| **1189a** | **Moisture**  **Content (%)** | **44.5**  **[42.1, 46.9] **** | **40.2**  **[37.8, 42.6] ***** | **25.4**  **[23.0, 27.8] ***** | **27.7**  **[25.3, 30.1] ***** | **42.6**  **[41.1, 44.2] **** | **35.3**  **[33.8, 36.9] ***** | **20.0**  **[18.5, 21.6] ***** | **14.4**  **[12.9, 16.0] ***** |  |
|  | **Weight**  **(10 grains in mg)** | **417**  **[396, 438]** | **452**  **[431, 473] *** | **480**  **[459, 501]** | **488**  **[467, 509]** | **473**  **[454, 492]** | **514**  **[494, 533] ***** | **476**  **[457, 496] **** | **506**  **[487, 525] ***** |  |
| **2316b** | **Moisture**  **Content (%)** | **42.2**  **[39.8, 44.6]** | **38.9**  **[36.2, 41.6] ***** | **27.1**  **[24.7, 29.5] ***** | **14.5**  **[12.1, 16.9] ***** | **42.0**  **[40.5, 43.6] **** | **30.4**  **[28.8, 31.9] ***** | **16.4**  **[14.8, 17.9] ***** | **9.6**  **[8.0, 11.1]** |  |
|  | **Weight**  **(10 grains in mg)** | **453**  **[432, 474] ***** | **482**  **[460, 504] ***** | **497**  **[476, 518] *** | **462**  **[441, 483]** | **464**  **[444, 483]** | **466**  **[446, 485] ***** | **431**  **[412, 450]** | **423**  **[404, 442]** |  |

**Table S2. Exome Capture Coverage and identified SNPs**

| **Sample** | **No. of Positions** | **Average**  **Coverage (%)** | **Positions**  **Interrogated**  **(QUAL ≥ 20)** | **Positions with**  **ambiguous alleles** | **No. of SNPs** | **Transitions**  **(G:A, C:T)** | **%** |
| --- | --- | --- | --- | --- | --- | --- | --- |
| **1189a Staygreen bulk** | 131890905 | 41.87 | 87436528 | 281007 | 63667 | 20368 | 32.0 |
| **1189a Non-SG bulk** | 131808060 | 39.72 | 86231450 | 289595 | 62153 | 19840 | 31.9 |
| **2316b Staygreen bulk** | 133560822 | 43.16 | 89672224 | 277857 | 66506 | 21240 | 31.9 |
| **2316b Non-SG bulk** | 131470978 | 35.80 | 80600195 | 257888 | 57320 | 18437 | 32.2 |
| **1189a Parent** | 131990025 | 42.66 | 88654268 | 285206 | 65787 | 21503 | 32.7 |
| **2316b Parent** | 134157857 | 43.12 | 89314438 | 278525 | 68215 | 22125 | 32.4 |
| **Paragon** | 133301196 | 39.05 | 85580450 | 269711 | 62916 | 20149 | 32.0 |

**Table S3. KASP primers for 1189a.** Primers were designed manually following extraction and alignment of flanking sequences to detect homoeologous and varietal differences. Primers for positions not listed in the BSA results reported in Table 2 are also listed and resulted from initial analysis of 1189a parental exome capture data.

| **Position** | **Primer Name** | **Paragon (Hex)** | **1189a Mutant (Fam)** | **Common** | **Cycling Conditions** |
| --- | --- | --- | --- | --- | --- |
| chr6A_25687659 | #61 | CCAGGGTAGTTATCCTTCTACc | CCAGGGTAGTTATCCTTCTACt | TTGTTGTGGAGCTGCGATA | 30-40 cycles 57/72°C |
| chr6A_46782541 | 6A#47 | GCAATGTGGTGTCTGCGACg | GCAATGTGGTGTCTGCGACa | TACGAGCTGCGCAAGCAGT | 30-40 cycles 60°C |
| chr6A_52406777 | 6A#55 | CAAATGACTGATGGGTACCTTc | CAAATGACTGATGGGTACCTTt | GCTAATGTTCCTTCCATAAGTG | 30-40 cycles 60°C |
| chr6A_55741222 | bsa#6A#1 | GTTGCAACAAAATTAGTCCACAAg | GTTGCAACAAAATTAGTCCACAAa | GTGATGCCAATTCTCAAAACG | 30-40 cycles 57/72°C |
| chr6A_57668862 | 6A#38 | CCTCGTCTATCTGGAATTTGg | CCTCGTCTATCTGGAATTTGa | ACGATGGTTTGAGTGGAGAG | 30-40 cycles 57°C |
| chr6A_66431084 | 6A#35 | AAAGGCATAAATCTCTCCATGg | AAAGGCATAAATCTCTCCATGa | CGCAATGACAGGTTTGTGCAA | 30-40 cycles 60/72°C |
| chr6A_67373981 | bsa#6A#2 | GCCTACGCCTTCGCCTTCg | GCCTACGCCTTCGCCTTCa | CATGGAGACGGCGAAGACC | 30-40 cycles 57/72°C |
| chr6A_76523438 | 6A#7 | GGTCGACCACCGGCTTCAc | GGTCGACCACCGGCTTCAt | CTCAACCCTTACCTCGAGGC | 30-40 cycles 57°C |
| chr6A_77099433 | bs#6A#3 | CTCGTGCATGATCCAGTTGg | CTCGTGCATGATCCAGTTGa | GTCAAGAAGGCGCTCGTCT | 30-40 cycles 57°C |
| chr6A_80699949 | 6A#19 | TACAAGTGTATGCCTTTGCTGg | TACAAGTGTATGCCTTTGCTGa | GTAGTAAGTGCTATTATATAACCTA | 30-40 cycles 60°C |
| chr6A_84072498 | bsa#6A#4 | GTAATCTTGCACCTATGTATAAg | GTAATCTTGCACCTATGTATAAa | GATCTAGCTAGCTTCTGCTG | 30-40 cycles 57°C |
| chr6A_93079117 | 6A#24 | ATGAACAACAGTCGCCCTGc | ATGAACAACAGTCGCCCTGt | ACTGTCGAAAGTTATTGACTGT | 30-40 cycles 57°C |
| chr6A_94068104 | bsa#6A#5 | AACACAAGAGATGGAGTGAGg | AACACAAGAGATGGAGTGAGa | AAGAAGGGGCAGTATTTTGATA | 30-40 cycles 57°C |
| chr6A_96601065 | bsa#6A#6 | CGCCATGACGGCGATCGAg | CGCCATGACGGCGATCGAa | CCGAAGTGTGGACGAGGATC | 30-40 cycles 57/72°C |
| chr6A_104309957 | 6A#30 | AAATGCAAGTGCTAGGTTGGg | AAATGCAAGTGCTAGGTTGGa | CTTCCTCTACACTCGGTTTCT | 30-40 cycles 57°C |
| chr6A_105559955 | 6A#1 | GGTCGACCACCGGCTTCAc | GGTCGACCACCGGCTTCAt | CTCAACCCTTACCTCGAGGC | 30-40 cycles 57°C |
| chr6A_116709274 | bsa#6A#8 | CTGAAGTAGCATGACAGCTGc | CTGAAGTAGCATGACAGCTGt | ACAATGCAAGTTCTATTGGTGT | 30-40 cycles 57°C |
| chr6A_142783784 | 6A#12 | GAGCCTGGAAGTATTGCTGc | GAGCCTGGAAGTATTGCTGt | CCTTGGACAAGCCAACAATC | 30-40 cycles 57/72°C |
| chr6A_379658960 | 6A#8 | AACAAAAGTCAACAGGTAACTAc | AACAAAAGTCAACAGGTAACTAt | GATGTCCCAAAGTTTACAAATC | 30-40 cycles 57°C |
| chr6A_431054756 | 6A#4 | TCAGCTCGCCACATAGTCAg | TCAGCTCGCCACATAGTCAa | GCACGATCACTCAACATCCT | 30-40 cycles 57°C |
| chr6A_459076102 | 6A#5 | CATCGCCCCGCGAAGCGg | CATCGCCCCGCGAAGCGa | GTCACCCGAAACCACCTGG | 30-40 cycles 57/72°C |
| chr6A_479267518 | 6A#14 | ACCTTCCTCAACCTGCCAAc | ACCTTCCTCAACCTGCCAAt | GAAACAGGCATGATCAGGAG | 30-40 cycles 57/72°C |
| chr6A_545816649 | 6A#GS2 | GCTTATGTCTTTGCGATCTGAg | GCTTATGTCTTTGCGATCTGAa | GACACACCAATGTGAGGAAAG | 30-40 cycles 57/72°C |
| chr6A_549531562 | 6A#GS3 | GGTGGGTGTAGTTCATCTCTc | GGTGGGTGTAGTTCATCTCTt | TCACAAACGAGCTTTTCTCGG | 30-40 cycles 57°C |
| chr6A_564876675 | #64 | ACATAGGAAGTGATATCTGTGg | ACATAGGAAGTGATATCTGTGa | AAGCAATCGTTCAAGTATGGC | Hydrocycler |
| chr6A_581749266 | 6A#GS5 | AAAGGCAATGCAGCCACCAc | AAAGGCAATGCAGCCACCAt | CCTAAAGAGATTGCATGGAAGT | 30-40 cycles 57°C |
| chr6A_585431913 | 6A#GS6 | CAGATACTGCATACTAGGATGg | CAGATACTGCATACTAGGATGa | AGGCGATTAGTAGTTTCACTC | 30-40 cycles 57°C |
| chr6A_591732057 | 6A#GS8 | TGGCCAGCATGATGGCGATg | TGGCCAGCATGATGGCGATa | CTCTACCTCGGCTACTCAC | 30-40 cycles 57°C |
| chr6A_603205645 | #63 | GTAAAGGACGGATTTATATACAGg | GTAAAGGACGGATTTATATACAGa | GTGATCATTCAAATGTGTCGGTG | Hydrocycler |

**Table S4. KASP primers for 2316b.** Primers were designed manually following extraction and alignment of flanking sequences to detect homoeologous and varietal differences.

| **Position** | **Primer Name** | **Paragon (HEX)** | **2316b Mutant (FAM)** | **Common** | **Cycling Conditions** |
| --- | --- | --- | --- | --- | --- |
| chr6D_36628312 | 2316b-6D#1 | CGAATTGACGCGATTTTTTCTc | CGAATTGACGCGATTTTTTCTt | GAGAGGAAAAGACGTCCGC | 30-40 cycles 57°C |
| chr6D_42295410 | 2316b-6D#13 | CAATAATTAATCCGGGTCCATg | CAATAATTAATCCGGGTCCATa | CAAAATCTGACCAGTTGATATG | 30-40 cycles 57°C |
| chr6D_50958137 | 2316b-6D#2 | GGCTTCAGCAGCAAATTCATAc | GGCTTCAGCAGCAAATTCATAt | CACTACTCCAGCAACTTCTTAG | 30-40 cycles 60°C |
| chr6D_57044844 | 2316b-6D#14 | GGTCACCCGGGCGAAATGg | GGTCACCCGGGCGAAATGa | CCCCCATCATCCGGGATGA | 30-40 cycles 60°C |
| chr6D_60487321 | 2316b-6D#15 | CGCTCGTGTTCTACCGCGg | CGCTCGTGTTCTACCGCGa | TCGGTGAGGCGGTATTCATG | 30-40 cycles 60°C |
| chr6D_65341694 | 2316b-6D#16 | GAAGGTGCTTAAGACGCTGg | GAAGGTGCTTAAGACGCTGa | CACTACCACATCATCACTGTC | 30-40 cycles 57°C |
| chr6D_72022762 | 2316b-6D#17 | GGATTGGTCTGCAAGAAAATAAg | GGATTGGTCTGCAAGAAAATAAa | TCAAATTTTGGTATGGAGCATTTT | 30-40 cycles 60°C |
| chr6D_79076045 | 2316b-6D#3 | GTGGAGGACACGATGGCAg | GTGGAGGACACGATGGCAa | GAGATAGAACGGGGTCCTG | 30-40 cycles 57°C |
| chr6D_84222670 | 2316b-6D#4 | GGAACTCAAGTTCAGGTAAAGg | GGAACTCAAGTTCAGGTAAAGa | ATCGGTCAGCTGAAAGCCC | 30-40 cycles 60°C |
| chr6D_86733052 | 2316b-6D#18 | CAATCTGCTTCCATGCTACGg | CAATCTGCTTCCATGCTACGa | AATGCAACTTTTACAGCACAAAAT | 30-40 cycles 60°C |
| chr6D_98263118 | 2316b-6D#5 | CACAGAATCTCCTCGGCGc | CACAGAATCTCCTCGGCGt | CAGAAACTAATTTGGACCACG | 30-40 cycles 57°C |
| chr6D_110872402 | 2316b-6D#6 | GCAACAGCTCTCCCATCGc | GCAACAGCTCTCCCATCGt | CCAAGGATGCAGCCTCAGC | 30-40 cycles 57°C |
| chr6D_119464714 | 2316b-6D#7 | GGTCCATCATTACAGGCCGc | GGTCCATCATTACAGGCCGt | TCGGCGCTCACAGCTGCAA | 30-40 cycles 60°C |
| chr6D_139310981 | 2316b-6D#8 | CCACTAGACAGGTATATACCc | CCACTAGACAGGTATATACCt | CTGCAACAGCCGTTCAAAAG | 30-40 cycles 57°C |
| chr6D_140102996 | 2316b-6D#9 | GTGTTATTCAGGGCGAAACGg | GTGTTATTCAGGGCGAAACGa | GGTCAACTGCTCTATGCTGC | 30-40 cycles 60°C |
| chr6D_148079666 | 2316b-6D#10 | GGACACCGACATGCCGGg | GGACACCGACATGCCGGa | TGCACGAGCGGATAGCCAA | 30-40 cycles 57°C |
| chr6D_264681596 | 2316b-6D#11 | TTTTTAGCAACTGCTGCATTGc | TTTTTAGCAACTGCTGCATTGt | GCTATTACCATTCACATCCTG | 30-40 cycles 57°C |
| chr6D_292379919 | 2316b-6D#12 | TGAAACCTGACGGAAGAAAGg | TGAAACCTGACGGAAGAAAGa | TCTATATACTGGCAAAGCAGC | 30-40 cycles 57°C |
| chr6D_352357451 | 2316b-6D#19 | GGTGGTCGGCGAGGCGAg | GGTGGTCGGCGAGGCGAa | CTTCGCCATCCTCCACCAC | 30-40 cycles 57°C |
| chr6D_363004043 | 2316b-6D#20 | CGCTGCGGTCCGAGGGg | CGCTGCGGTCCGAGGGa | CTGGGCCCCGCACGTGA | 30-40 cycles 57°C |
